# Hypermethylation of mitochondrial DNA in vascular smooth muscle cells impairs cell contractility

**DOI:** 10.1038/s41419-020-2240-7

**Published:** 2020-01-20

**Authors:** Yue-Feng Liu, Juan-Juan Zhu, Xiao Yu Tian, Han Liu, Tao Zhang, Yun-Peng Zhang, Si-An Xie, Ming Zheng, Wei Kong, Wei-Juan Yao, Wei Pang, Chuan-Rong Zhao, Yuan-Jun Tang, Jing Zhou

**Affiliations:** 1 Department of Physiology and Pathophysiology, School of Basic Medical Sciences, Peking University, Beijing, People’s Republic of China; 2 Key Laboratory of Molecular Cardiovascular Sciences, Ministry of Education, Beijing, People’s Republic of China; 3 School of Biomedical Sciences, Institute of Vascular Medicine, CUHK Shenzhen Research Institute, Chinese University of Hong Kong, Hong Kong, People’s Republic of China; 4 Department of Vascular Surgery, Peking University People’s Hospital, Beijing, People’s Republic of China

**Keywords:** Vascular diseases, Energy metabolism

## Abstract

Vascular smooth muscle cell (SMC) from arterial stenotic-occlusive diseases is featured with deficiency in mitochondrial respiration and loss of cell contractility. However, the regulatory mechanism of mitochondrial genes and mitochondrial energy metabolism in SMC remains elusive. Here, we described that DNA methyltransferase 1 (DNMT1) translocated to the mitochondria and catalyzed D-loop methylation of mitochondrial DNA in vascular SMCs in response to platelet-derived growth factor-BB (PDGF-BB). Mitochondrial-specific expression of DNMT1 repressed mitochondrial gene expression, caused functional damage, and reduced SMC contractility. Hypermethylation of mitochondrial D-loop regions were detected in the intima-media layer of mouse carotid arteries subjected to either cessation of blood flow or mechanical endothelial injury, and also in vessel specimens from patients with carotid occlusive diseases. Likewise, the ligated mouse arteries exhibited an enhanced mitochondrial binding of DNMT1, repressed mitochondrial gene expression, defects in mitochondrial respiration, and impaired contractility. The impaired contractility of a ligated vessel could be restored by ex vivo transplantation of DNMT1-deleted mitochondria. In summary, we discovered the function of DNMT1-mediated mitochondrial D-loop methylation in the regulation of mitochondrial gene transcription. Methylation of mitochondrial D-loop in vascular SMCs contributes to impaired mitochondrial function and loss of contractile phenotype in vascular occlusive disease.

## Introduction

Vascular smooth muscle cells (SMCs) that constitute the majority of contractile cells of blood vessels, are responsible for maintaining vascular homeostasis through active contraction and relaxation. In vascular stenotic-occlusive diseases such as atherosclerosis and post-injury restenosis, SMCs undergo a switch from contractile/differentiated to synthetic/dedifferentiated phenotype, during which the contraction function of SMC is inhibited^1–3^. SMC contraction depends on energy provided by adenosine triphosphate (ATP) mainly generated through oxidative phosphorylation in the mitochondrion, the cellular powerhouses, or through anaerobic glycolysis. Previous study on arteries from rat, rabbit, dog, and pig indicated that SMC contraction is dependent rather exclusively on ATP derived from mitochondrial respiration^4^. Mitochondrial function is therefore crucial for normal function of SMCs. Mitochondrial dysfunction indicated by reduced mitochondrial DNA (mtDNA) copy number and decreased mitochondrial oxygen consumption rate (OCR), is present in human atherosclerotic SMCs^5^. These evidence suggested the association between mitochondrial abnormality and loss of contractile phenotype in SMC during the development of vascular diseases.

Up to date, among all the 1500 mitochondrial proteins, 13 of which are encoded solely by mtDNA^6^. These 13 proteins are part of the constitute respiratory complexes I, III, and IV and the ATP synthase complex V; which are components of the oxidative phosphorylation system^6^. In each mitochondrion, mtDNA in the form of a multicopy, 16569-bp circular double-stranded DNA is associated with the mitochondrial inner membrane. Mutations and deletions in mtDNA or mitochondrion-related nuclear DNA genes have been indicated in mitochondrial dysfunction^7^. Suppression of mtDNA transcription results in gradual loss of oxidative phosphorylation, ATP production, and energy-dependent functions, such as cell contractility^8^. Mitochondrial reactivation by overexpressing the mitochondrial helicase Twinkle, an mtDNA polymerase, has been effective for treatment of atherosclerosis in mouse model^5^. Pharmaceutical rescue of mitochondrial function also prevents SMC proliferation in vitro^9^. However, the causes and mechanisms of mitochondrial gene suppression especially in SMCs are still unclear.

MtDNA contains a unique 1124-bp non-coding region, which is known as the displacement loop (D-loop). D-loop is the control region of mtDNA replication and also as promoters of mitochondrial gene transcription^10^. Methylated cytosines have been found within the human D-loop from cultured cell and tissues using methods such as methylation-specific-polymerase chain reaction (MSP), bisulfite sequencing, and methylated DNA immunoprecipitation^11,12^. However, the presence of this epigenetic modification in mitochondrion has been challenged due to conflicting findings^13,14^. Intriguingly, several studies showed the presence of DNA methyltransferases (DNMTs), mainly DNMT1 and DNMT3A, inside mitochondria in support of the occurrence of methylation^15–17^.

In the present study, we hypothesized that the maintenance DNA methyltransferase DNMT1 are present in the mitochondrial D-loop in vascular SMCs and their accumulation in mitochondria is increased in response to vascular stress; hypermethylation of mitochondrial D-loop region suppresses mitochondrial gene transcription and function, reprograms mitochondrial metabolism, leading to defects in cell contractility and aberrant cell growth.

## Materials and methods

Additional methods are available in the online-only Data Supplement.

### Cell culture

Primary human umbilical artery smooth muscle cells (SMCs) were isolated from human umbilical arteries. SMCs were maintained in Nutrient Mixture F12 Ham Kaighn’s Modification (F12K, Sigma Aldrich) supplemented with 20% (for cell maintaining) or 2% (for treatment) fetal bovine serum (FBS) (Gemini) and 10% SMC Growth Medium (Cell Applications). Mouse embryonic fibroblasts (MEFs) were isolated from Dnmt1^flox/flox^ mice embryo and were maintained in dulbecco’s modified eagle medium (DMEM) (Gibco) supplemented with 10% fetal bovine serum (FBS) (Gemini).

### Viruses and plasmids

Ad-shDNMT1 carrying short-hairpin RNA (shRNA) specifically targeting DNMT1 and the control adenovirus expressing GFP (Ad-GFP) were obtained from Vigene Biosciences. Adenovirus expressing the Cre recombinase (Ad-Cre) was obtained from SignaGen. pcDNA3/Myc-DNMT1 (no-MTS-DNMT1), in which the full-length cDNA for human DNMT1 was cloned into EcoRI and NotI sites of pcDNA3/Myc, was a gift from Arthur Riggs (Addgene plasmid # 36939). pDsRED2-Mito plasmid, in which the mitochondria targeting sequence (MTS) is fused to the 5′-end of pDsRed2, was from Clontech. The MTS-DsRED2 fragment was amplified by primer sets (forward primer: 5′-TCAGAGGAGGACCTGGAATTCATGTCCGTCCTGACGCCGC-3′ reverse primer: 5′-ACCACCTGTTCCTGTAGGAATTCATGCCGGCGCGTACC-3′) with the pDsRED2-Mito plasmid served as the template. The fragment was cloned into 939 sites of pcDNA3/Myc-DNMT1, upstream of DNMT1 open-reading frame (ORF), to generate MTS-DNMT1. Validity of the MTS-DNMT1 construct was verified by base sequencing.

### Traction force microscopy (TFM)

Polyacrylamide (PA) gel substrates were prepared by mixing acrylamide, bis-acrylamide, and fluorescence beads with a diameter of 0.2 μm (Thermo Fisher), ammonium persulfate and tetramethylethylenediamine in ultrapure water. The mixture was added to glass-bottomed dishes and then the gel surfaces were activated and were then coated with fibronectin. SMCs were seeded on the gel substrates. A spatial map for each dish of fluorescent beads that were embedded within the gel substrate directly underneath the cells was taken by a fluorescence microscope (Leica DMI6000B). Following detachment of cells from the substrates using 0.5% trypsin, a second spatial map of the same beads was obtained. Monolayer displacement was calculated by comparing the two maps using a Fourier-based difference-with-interpolation image analysis^18^. To characterize the contractile forces of each cell, the elastic strain energy stored in gels due to cell tractions was calculated as the product of local tractions and deformations, integrated over the spreading area of the cells^19^.

### Mitochondria transplantation in vitro and ex vivo

Mitochondrial transplantation in vitro was performed as previously described^20^. In brief, the donor cells were trypsinized, washed, and ruptured. The cell homogenate was blended and centrifuged for 5 min at 1500 × *g*. The supernatant was then added into a new centrifuge tubes and centrifuged for 1 min at 15,000 × *g*. The mitochondria pellets were washed and resuspended in the appropriate incubation medium. The whole purification process was performed on ice or at 4 °C. The recipient cells were grown to 70–80% confluence and were then cultured in F12 media supplemented with 110 µg/mL sodium pyruvate, 50 µg/mL uridine, and 100 ng/mL ethidium bromide to remove endogenous mitochondria. Fourteen days later, mtDNA copy number and cell immunofluorescence were assessed in the recipient cells to verify the efficiency of mitochondria-removal. The recipient cells were then incubated with 2.35 × 10^8^/mL of isolated mitochondria from control, PDGF-BB treated or MTS-DNMT1-transfected SMCs in an incubator at 37 °C for 24 h. For mitochondria delivery ex vivo, mouse arterial rings (3 mm) were incubated in mitochondrial suspensions (isolated mitochondria (2.35 × 10^8^/mL) in DMEM medium) for 12 h.

### Experimental animals

All animal studies were performed in accordance with the approved protocol (LA2015017) of the Animal Care and Use Committee of Peking University and were performed in accordance with the “Guide for the care and use of laboratory animals” published by the US National Institutes of Health (publication No. 85–23, revised 1996). Eight-week-old C57/BL6 wild-type male and female mice were obtained from the Experimental Animal Center at Peking University Health Science Center (Beijing, China). Dnmt1^flox/flox^ (B6.129S4-Dnmt1^tm2Jae^/Mmucd, stock number 014114-UCD) mice were obtained from the Mutant Mouse Resource and Research Center (MMRRC) at the Jackson Laboratory. Animals were kept in specific pathogen-free cages, 12-h light–dark cycle, controlled temperature and humidity, and had water and food ad libitum. Anesthetization and euthanasia were performed by intraperitoneal injection of sodium pentobarbital (50 mg/kg and 150 mg/kg, respectively).

### Human specimens

Endarterectomy specimens and internal mammary arteries were obtained from patients undergoing coronary artery bypass grafting or with carotid occlusive diseases. All samples were obtained with the agreement of the patients and approved by the Peking University People’s Hospital Medical Ethics Committee (2015PHB024). The experiments using human specimens were carried out in accordance with the approved guidelines. All human studies described in this work conform to the principles outlined in the Declaration of Helsinki.

### Arterial respiration

Oxygraph-2k (O2k; OROBOROS Instruments) was used for measuring mitochondria respiration in mouse arteries. Mouse common carotid arteries were dissected and maintained at 4 °C. Samples were permeabilized and were then washedbefore being put into the electrode chambers. Substrates and inhibitors were added sequentially to determine complex I, II, and IV respiration as indicated in a previous study^5^. Complex I-supported respiration rates were measured by using 10 mmol/L glutamate + 5 mmol/L malate. 5 mmol/L ADP was then added to stimulate State 3 respiration. After the addition of 1 µmol/L rotenone for the inhibition of complex I, complex II-supported respiration was assessed with 10 mmol/L succinate. Next, Complex III-supported respiration was inhibited by 5 µmol/L antimycin, and then 0.5 mmol/L N,N,N′,N′-Tetramethyl-p-phenylenediamine (TMPD) + 2 mmol/L ascorbate were used to induce complex IV-supported respiration. The intactness of the outer mitochondrial membrane was assessed by adding 10 µmol/L cytochrome C finally. The platform oxygen flux (pmol/s) difference was measured by Oxygraph-2k after every reagent was added into chambers. Vessels were removed from the electrode chambers and dried, with oxygen flux expressed as picomoles O_2_ per second per dry weight (pmol/s/mg).

### Wire myograph

Wire myograph experiment was performed as previously described^21^. In brief, Mice were euthanized and common carotid arteries were harvested. The arteries with or without mitochondrial transplantation were placed in ice-cold Krebs solution with oxygen. Common carotid arteries were cleaned of adhering tissue and cut into ring segments of 3 mm in length, and then the ring segments were suspended in the myograph (Danish Myo Technology, Aarhus, Denmark). To monitor the changes in isometric tension, KCl-simulated and phenylephrine-simulated vascular contraction were expressed as active tension. We set the contraction baseline at about 1.5 mN, the active tension was the difference value between simulated contraction and basal contraction per unit ring length (mN/mm).

### Statistics

Data are expressed as mean ± SEM from at least three independent experiments. Results were analyzed by SPSS and GraphPad Prism 7.0 software for statistical significance between treatment groups. Normality and equal variance tests were confirmed before further analysis. Parametric data with equal variance were analyzed by paired or unpaired two-tailed Student’s *t*-test. Nonparametric data were analyzed using Mann–Whitney *U*-test. Multiple comparisons of means were analyzed using two-way ANOVA followed by Tukey’s post hoc test. Values of *P* < 0.05 were considered statistically significant.

## Results

### DNMT1 translocate to the mitochondria and catalyze D-loop methylation in response to PDGF-BB

**Fig. 1:**
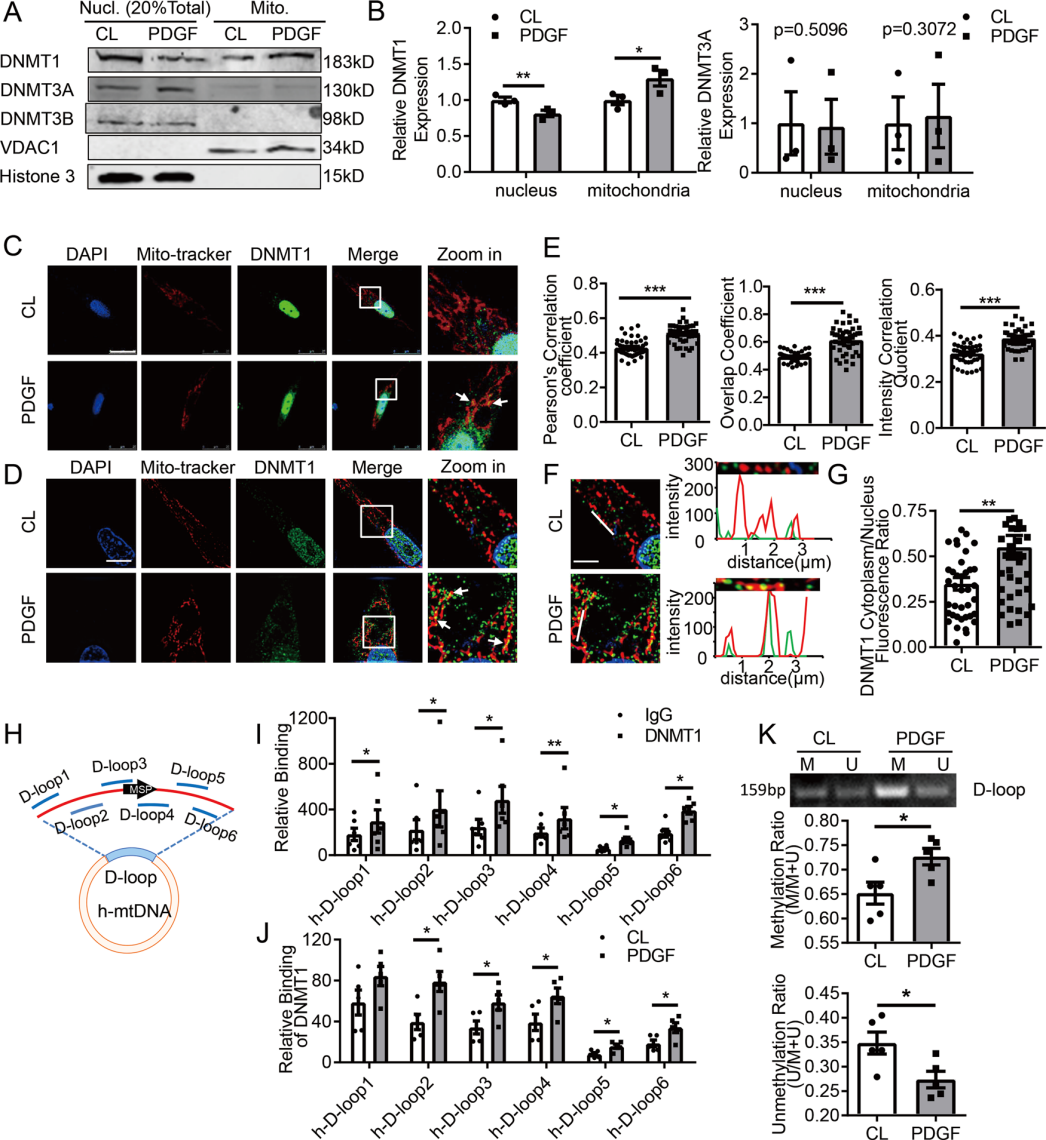
DNA methyltransferase 1 (DNMT1) undergo a nucleus-to-mitochondrial translocation in PDGF-BB-stimulated vascular smooth muscle cells. Vascular SMCs were subjected to vehicle (CL) or PDGF-BB (20 ng/mL) treatments for 24 h, and **a** accumulations of DNMT1, -3A, and -3B in nuclear (Nucl.) and mitochondrial (Mito.) fractions were analyzed (*n* = 3). Shown are representative blots. **b** Semi-quantification of DNMT1 and -3A in A. Expression was normalized to the respective internal reference proteins (VDAC1 or Histone 3) and expression in CL cells was set as 1. **c**, **d** Subcellular localization of DNMT1 was assessed by immunofluorescent staining followed by confocal microscopy (**c**) or structured illumination microscopy (**d**). Mito-tracker illustrates mitochondria. Arrowheads indicated co-localizations. Scale bar: 25 μm for c and 10 μm for **d**. **e** Co-localization of DNMT1 with mitochondria (mito-tracker) were quantified. Cells were randomly selected in microscopic fields from five biological replicates (*n* = 45). **f** Fluorescence intensity profiles indicate the degree of overlap between DNMT1 and mitochondria. Scale bar: 25 μm. **g** Ratio of fluorescence intensity of DNMT1 in cytoplasm over nucleus was measured (*n* = 40). **h** Schematic diagram of human mitochondrial DNA (h-mtDNA). Locations of primer sets for chromatin immunoprecipitation (ChIP) and methylation-specific PCR (MSP) were indicated. **i** DNMT1 bindings to D-loop regions in cells with CL treatment were analyzed by ChIP assay (*n* = 5). **j** DNMT1 bindings to D-loop regions in CL- and PDGF-BB-treated cells were analyzed (*n* = 5). **k** Methylation status of the D-loop region was measured by MSP (*n* = 5). M: methylated; U: unmethylated. *P < 0.05 **P < 0.01 ***P < 0.005 by student’s *t*-test (**b**, **i**, **j**, and **k**) and Mann–Whitney test (**e**, **g**), Error bars show ± SEM.

Platelet-derived growth factor-BB (PDGF-BB)is a major growth factor to stimulate SMC proliferation and phenotypic change contributing to the development of vascular stenotic-occlusive diseases^22^. Treatment of cultured human umbilical arterial SMCs with PDGF-BB at 20 ng/mL for 24 h induced expressions of genes related to cell cycle progressive and proliferation while inhibited expressions of contractile genes (Fig. S1). Immunoblots of nuclear versus mitochondrial fractions showed DNMT1 and DNMT3A proteins exist both in nuclei and mitochondria (Fig. 1a), whereas DNMT3B were found exclusively in the nuclei (Fig. 1a), indicated by nuclear marker Histone 3 and mitochondrial marker VDAC1 (Fig. 1a). PDGF-BB did not alter DNMT1 expression (Fig S2). However, PDGF-BB treatment increased DNMT1 accumulation in the mitochondria with a decrease in the nuclei (Fig. 1b). Structured illumination microscopy (SIM) demonstrated increased translocation of DNMT1 from cytosol to mitochondria in response to PDGF-BB, comparing to little mitochondrial co-localization of DNMT1 under basal condition (Fig. 1c–g). These data suggest a mitochondrial translocation of DNMT1 upon PDGF-BB stimulation, accompanied by the loss of contractile phenotype.

To study the function of mitochondrial DNMT1 in SMC, we first examined DNMT1 association with D-loop using chromatin immunoprecipitation (ChIP) assay. Six overlapping PCR primer sets were designed to span the 16,031 to 604 regions of human mtDNA (Fig. 1h). Immunoprecipitates from anti-DNMT1 antibody were enriched for D-loop mtDNA in comparison with that from IgG (Fig. 1i). DNMT1 association with D-loop regions was enhanced in PDGF-BB-treated cells comparing to vehicle (Fig. 1j). Methylation-specific PCR (MSP) assay using primers targeting the D-loop regions (Fig. 1h) indicated an increase of mtDNA methylation level in PDGF-BB-treated cells (Fig. 1k and Fig. S3). In addition, inhibition of DNMTs by 5-Aza-2′-deoxycytidine, or by adenoviral DNMT1-shRNAs both suppressed D-loop methylation (Fig. S4A, B). Taken together, these results suggest that the PDGF-BB induced DNMT1 is responsible for D-loop methylation in SMC mitochondria.

### Mitochondrial-specific targeting DNMT1 repress mitochondrial gene expression

**Fig. 2:**
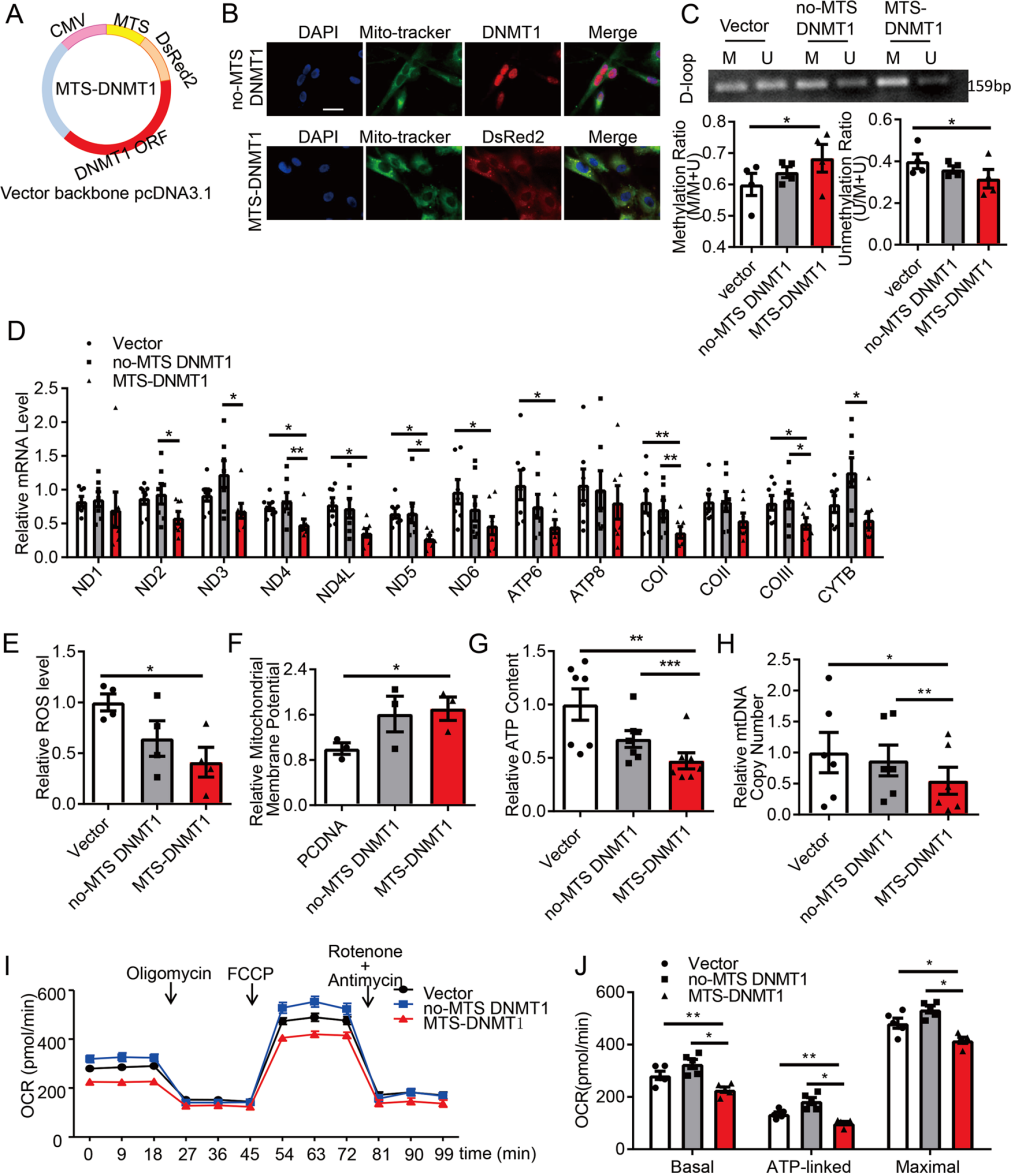
Expression of exogenous mitochondrial-targeting DNMT1 results in hypermethylation of D-loop and compromises mitochondrial function. **a** Schematic diagram of the MTS-DNMT1 construct. MTS mitochondrial-targeting sequence, CMV cytomegalovirus promoter, ORF open-reading frame. **b** Representative immunofluorescent staining of mitochondria (Mito-tracker), no-MTS-DNMT1, and MTS-DNMT1 (DsRed2, Discosoma sp. red fluorescent protein 2). Cells were transfected either with no-MTS-DNMT1 (upper) or with MTS-DNMT1 (lower). Scale bar: 75 μm. **c**–**j** Vascular SMCs were transfected with empty vectors (pcDNA-3.1), no-MTS DNMT1, or MTS-DNMT1, and **c** The methylation status of D-loop region was measured by Methylation-specific PCR (MSP). (*n* = 4). **d** The mRNA levels of mtDNA genes were detected by quantitative RT-PCR (*n* = 7). **e** ROS production was detected by flow cytometry (*n* = 4); **f** Mitochondrial membrane potential was measured by flow cytometry (*n* = 3); **g** Intracellular ATP content was assessed by a luminometer (*n* = 7); **h** MtDNA copy number was measured by quantitative RT-PCR assay (*n* = 6); **i** Representative traces for oxygen consumption rates (OCR), which were assayed using a Seahorse XF24 flux analyzer, with sequential injections of mitochondrial effectors at time points indicated by the arrows (*n* = 5); **j** The basal, ATP-linked, and maximal OCR in I were analyzed. ND NADH dehydrogenase, ATP ATP synthase, CO cytochrome c oxidase, Cytb Cytochrome b, ROS reactive oxygen species, M methylated, U unmethylated, FCCP carbonyl cyanide 4(trifluoromethoxy) phenylhydrazone *P < 0.05 **P < 0.01 ***P < 0.005 by one-way ANOVA with Tukey’s post hoc analysis. Error bars show ± SEM.

To further study the functional importance of DNMT1 and mtDNA methylation, we constructed plasmids expressing human full-length DNMT1 with an N-terminal mitochondrial-targeting sequence (MTS-DNMT1) to specifically target mitochondria (Fig. 2a). Comparing to DNMT1 proteins without MTS, which showed a predominant nuclear localization (Fig. 2b upper), MTS-DNMT1 were almost exclusively present in the mitochondria (Fig. 2b lower). MTS-DNMT1 increased D-loop region methylation (Fig. 2c). As a result, transcriptions of genes exclusively encoded by mtDNA, including NADH dehydrogenase 4 (ND4), ND4L, ND5, ND6, ATP synthase 6 (ATP6), cytochrome c oxidase I (COI), and COIII were downregulated by MTS-DNMT1 expression, while the nuclear localized DNMT1 without MTS has no effect on either mtDNA transcription (Fig. 2d), or the nuclear DNA-coded mitochondrial proteins such as VDAC1, Mitochondrial Calcium Uniporter (MCU), Mitochondrial Calcium Uniporter Regulator 1 (MCUR1), and Mitochondrial Calcium Uptake 1 or 2 (MICU1 and MICU2) (Fig. S5). Altogether, these data demonstrate that expression of mitochondrial-targeting DNMT1 results in hypermethylation of D-loop accompanied with an impairment of mtDNA transcription.

### Mitochondrial-specific targeting DNMT1 cause functional damage to the mitochondria

MtDNA-encoded proteins are crucial components of mitochondrial respiration complexes and energy metabolism^6^. We found that expression of MTS-DNMT1 caused decreased reactive oxygen species (ROS) production and intracellular ATP level, as well as increased mitochondrial membrane potential, and that expression of no-MTS DNMT1 did not (Fig. 2e–g). Since D-loop region has been suggested to regulate the mtDNA duplication, we detected mtDNA copy number and found that it was reduced in cells transfected with MTS-DNMT1 (Fig. 2h). Mitochondrial respiration was also determined using a seahorse extracellular flux analyzer. The MTS-DNMT1-transfected cells showed much lower basal, ATP-linked, and maximal oxygen consumption rate (OCR) (Fig. 2i, j). Of interest, there is an increase (but not significant) in the basal, ATP-linked and maximal OCR in cells with no-MTS DNMT1 transfection compared with that in the control cells (Fig. 2i, j), both supporting the role of DNMT1 on mitochondrial metabolism although the role of nuclear DNMT1 cannot be excluded.

### Mitochondrial-specific targeting DNMT1 reduces cell contractility

**Fig. 3:**
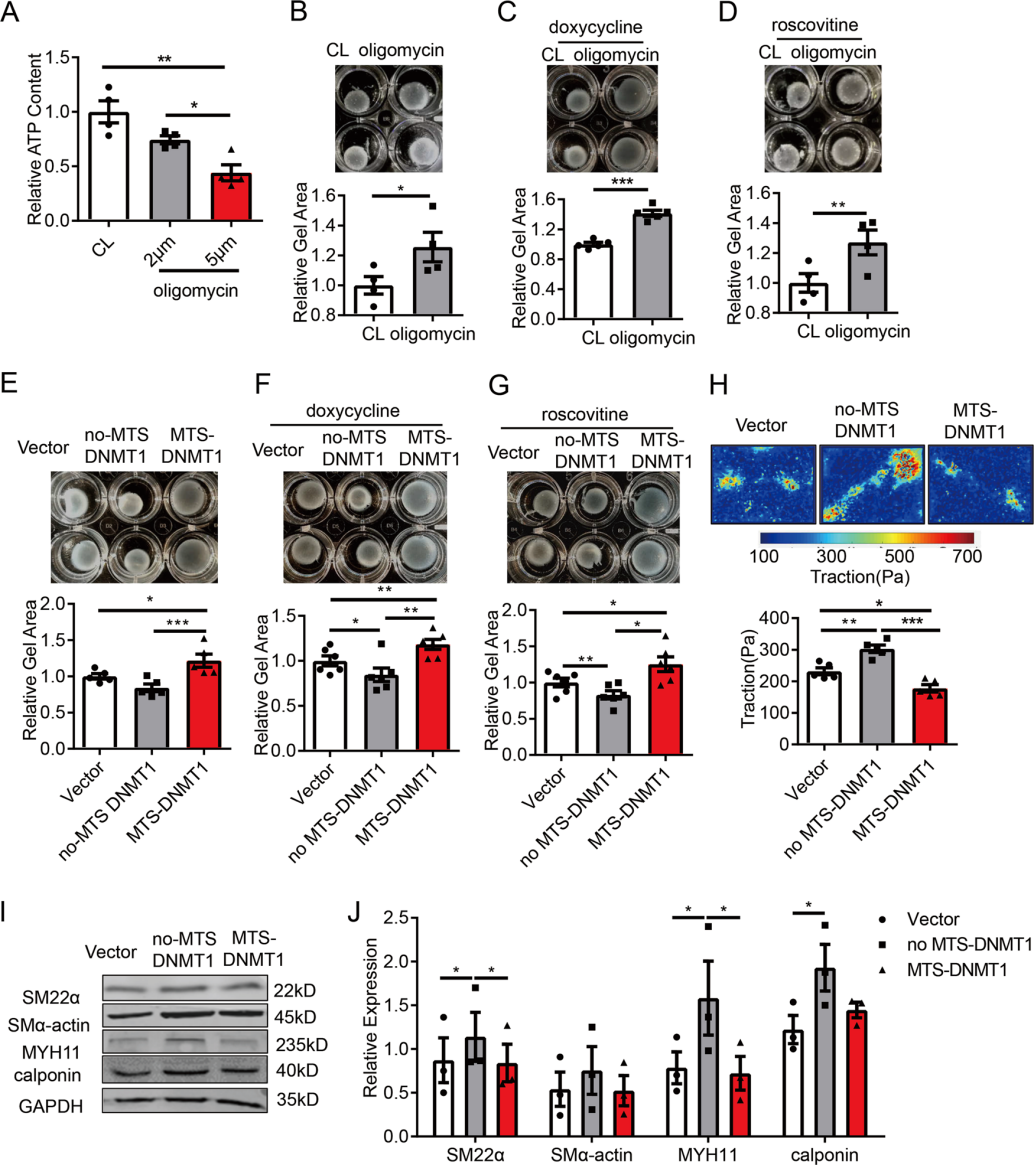
Expression of exogenous mitochondrial-targeting DNMT1 impairs smooth muscle cell contractility. **a** Vascular SMCs were treated with control (CL) or oligomycin (2 or 5 μmol/L) for 12 h and intracellular ATP content was assessed by a luminometer (*n* = 4). **b**–**d** Cells were embedded in collagen gel and were then treated with control (CL) or oligomycin (5 μmol/L) for 12 h in the absence (**b**) (*n* = 4) or presence of doxycycline (1 μg/mL) (**c**) or roscovitine (10 μmol/L) pre-treatment (12 h) (*n* = 5) (**d**). Cell contractions were determined by measuring the gel areas (*n* = 4). **e**–**g** Cells were transfected with empty vectors, no-MTS DNMT1, or MTS-DNMT1 and were then subjected to gel contraction assay in the absence (E) (*n* = 5) or presence of doxycycline (1 μg/mL) (**f**) or roscovitine (10 μmol/L) pre-treatment (12 h) (*n* = 6). **h** Single-cell contractility of SMCs was measured by traction force microscopy (TFM). Upper: Representative cell force images. Lower: Quantification of traction force with respect to the indicated treatments (*n* = 5). Scale bar: 25 μm. **i** Vascular SMCs were transfected with empty, no-MTS DNMT1, or MTS-DNMT1, and expressions of SMC contractile marker proteins were analyzed by western blot assay. Shown are representative blots (*n* = 3). **j** Semi-quantification of proteins in **i**. *P < 0.05, **P < 0.01, ***P < 0.005 by student’s *t*-test (**b**–**d**) and one-way ANOVA with Tukey’s post hoc analysis (**a**, **e**, **f**–**h**, **j**). Error bars show ± SEM.

To examine the role of DNMT1 on cell contractility, we first showed that treatment with oligomycin at 2 or 5 μmol/L to inhibit mitochondrial ATP synthase decreased intracellular ATP content by 24% and 56%, respectively (Fig. 3a). Gel contraction assay showed that cells treated with oligomycin at 5 μmol/L covered significant larger area of the cell-seeded collagen gels (Fig. 3b), suggesting an insufficient cell contractility. To exclude the influence of cell migration and proliferation on gross gel contraction, we used doxycycline (an inhibitor of matrix metalloproteinases) to inhibit migration and roscovitine (an inhibitor of CDKs) to inhibit cell proliferation (Fig. 3c, d), which did not show any effect, indicating that the reducing ATP contents impairs SMC contractility.

Expression of MTS-DNMT1 resulted in a largest gel area compared with control vector or the no-MTS DNMT1 in the absence or presence of doxycycline/roscovitine, indicating reduced contractility (Fig. 3e–g). Additionally, expression of no-MTS DNMT1 increased cell contractility (Fig. 3e–g). We then used traction force microscopy (TFM) to examine cell contractility in terms of the mechanical interplay between a single adherent cell and its substrate^23^. Cells expressing no-MTS DNMT1 generated greater traction forces whereas the cells expressing MTS-DNMT1 produced less (Fig. 3h). Contraction of vascular SMCs requiring a high rate of ATP consumption are accompanied by a transient increase in cytoplasmic free Ca^2+^ concentration. In SMCs treated with KCl to induce depolarization-dependent Ca^2+^ influx, no-MTS-DNMT1 increased intracellular Ca^2+^concentration, while MTS-DNMT1 reduced the intracellular Ca^2+^concentration measured by time-lapse imaging of Ca^2+^ probe fura-4 AM (Fig. S6), indicating that DNMT1-dependent methylation was also involved in Ca^2+^ signaling during SMC contraction.

Genes involved in SMC contractile function, including SM22α, MYH11, and calponin were upregulated in cells transfected with no-MTS DNMT. No detectable changes were found in cells expressing MTS-DNMT1 in comparison with the vector transfection (Fig. 3i, j), suggesting a diverse role of nuclear and mitochondrial DNMT1 in regulating contractile gene expressions. Proliferative SMCs have compromised cell contractility^24^. We detected SMC proliferation using Ki67 immunofluorescent staining. As expected, MTS-DNMT1 increased the ratio of Ki67-positive cells (Fig. S7). The cell death-related phenotypes were observed neither in cells with the above treatments nor in cells with inhibition of global DNMT1 (Figs. S8 and S9).

### Mitochondrial transplantation with healthy mitochondria restores smooth muscle cell contractility in vitro

**Fig. 4:**
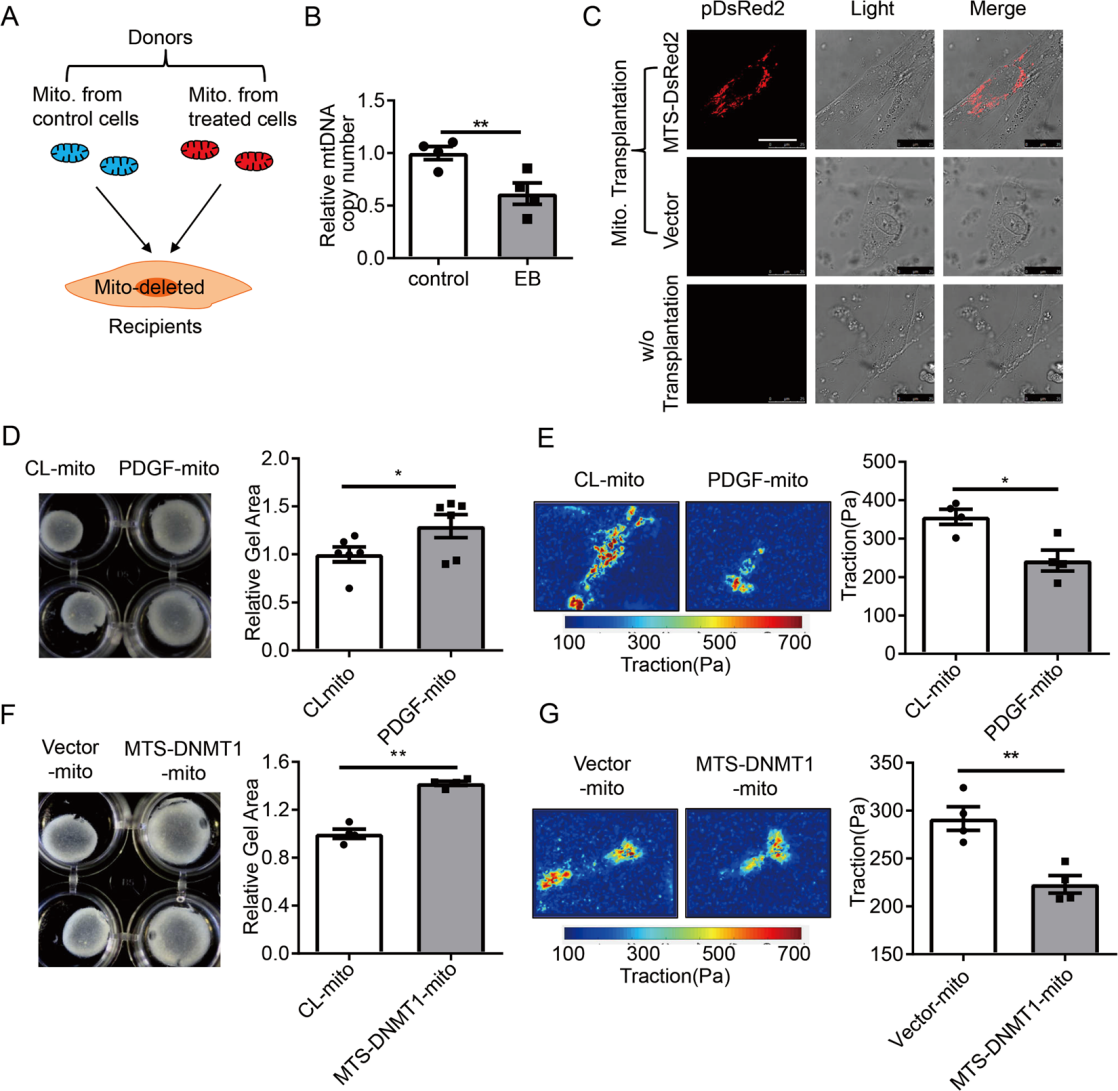
Mitochondrial transplantation restores smooth muscle cell contractility. **a** Schematic diagram for the process of mitochondrial transplantation. Mito. mitochondria. Mito-deleted cells were incubated with mitochondria prepared from the donor cells. Some donor cells were subjected to PDGF-BB or the vehicle treatments, others were transfected with MTS-DNMT1 plasmids or empty vectors. **b** Cells were treated with ethidium bromide (100 ng/mL) supplemented with sodium pyruvate (110 µg/mL), uridine (50 µg/mL) for 14 days to achieve mitochondrial deletion, and mtDNA copy numbers were detected by quantitative RT-PCR (*n* = 4). **c** Live-cell confocal imaging for cells receiving mitochondria from the donors. The donors were transfected with MTS-DsRed2 plasmids (upper) or with empty vectors (middle). Images for cells undergoing no transplantation were also captured (lower). Scale bar: 25 μm. **d**, **e** Gel contraction assay (*n* = 6) and traction force microscopy (*n* = 4) in cells receiving mitochondria from PDGF-BB- (PDGF-mito) or CL-treated (CL-mito) cells. Scale bar: 25 μm. **f**, **g** Gel contraction assay (*n* = 4) and traction force microscopy (*n* = 4) in cells receiving mitochondria from MTS-DNMT1 (MTS-DNMT1-mito) or control vectors (Vector-mito) transfected cells. Scale bar: 25 μm. *P < 0.05, **P < 0.01 by student’s *t*-test. Error bars show ± SEM.

To further investigate whether the reduction in SMC contraction is attributable to mitochondrial dysfunction, we performed mitochondrial transplantation experiments as described previously^20,25^. Mitochondria isolated from donor cells were transplanted into the mitochondria-deleted recipient cells (Fig. 4a). The efficiency of mitochondrial deletion with ethidium bromide was determined by examining the mtDNA level, which was decreased by appropriately 40% (Fig. 4b). Verification of exogenous mitochondria entry into recipient cells was performed by detecting the fluorescently labeled donor mitochondria in the recipients (Fig. 4c). Compared with recipient cells taken up mitochondria from cells with control treatment, cells receiving the PDGF-BB-treated mitochondria exhibited reductions both in gel contraction ability and in traction force (Fig. 4d, e). Moreover, cells receiving mitochondria from donors expressing MTS-DNMT1 showed more reduction in contractility in comparison with cells receiving mitochondria from donors with vector transfection (Fig. 4f, g).

### Mitochondrial D-loop region is hypermethylated in arterial SMCs from vascular stenotic-occlusive diseases

**Fig. 5:**
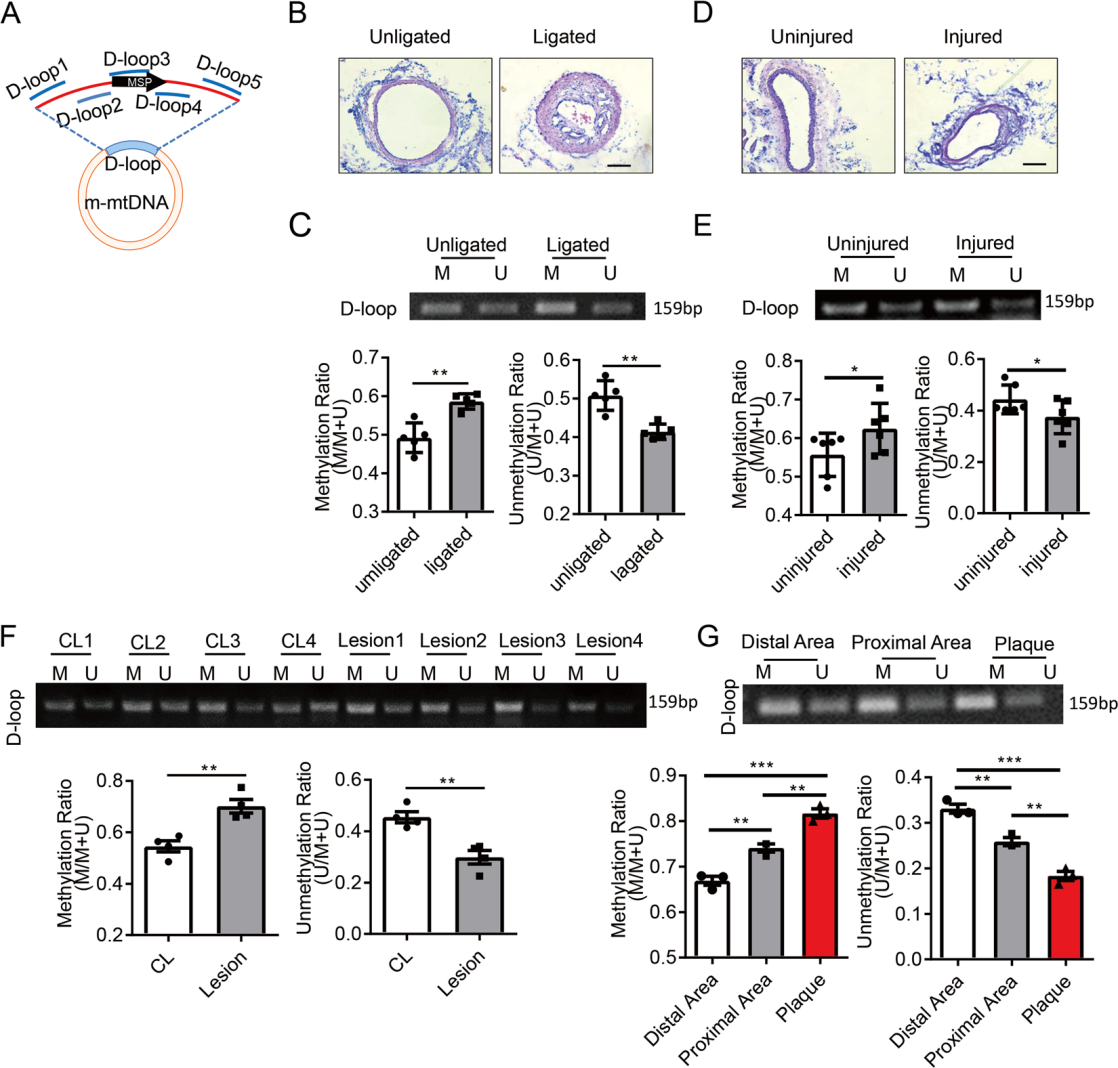
Mitochondrial D-loop region is hypermethylated in smooth muscle cells from arterial stenotic and occlusive diseases. **a** Schematic diagram of mouse mitochondrial DNA (m-mtDNA). Locations of primer sets for methylation-specific PCR (MSP) and chromatin immunoprecipitation (ChIP) were indicated. **b** Representative H&E staining of completely ligated and unligated mouse carotid arteries. Vessels were isolated 1 (for MSP) or 4 (for H&E staining) weeks after the surgery. Scale bar: 100 μm. **c** MSP to assay D-loop methylation in the isolated vessels using primers indicated in A and quantification of the MSP results (*n* = 5). Scale bar: 100 μm. **d** Representative H&E staining of guide-wire-injured carotid arteries and the uninjured control arteries. Vessels were isolated 4 weeks after the surgery. **e** MSP to assay D-loop methylation in the isolated vessels and quantification of the MSP results (*n* = 6). **f** Methylation status of D-loop region in endarterectomy specimens (lesion) and internal mammary arteries (CL) was assessed by MSP (*n* = 4). **g** Methylation status of D-loop region in human endarterectomy specimens from patients with carotid occlusive diseases was assessed by MSP (*n* = 3). Human endarterectomy specimens were dissected into three parts (distal area, proximal area and plaque) according to the degree of the lesion. M methylated, U unmethylated. *P < 0.05, **P < 0.01 by student’s *t-*test (**c**, **e**, and **f**) and one-way ANOVA with Tukey’s post hoc analysis (**g**). Error bars show ± SEM.

We next examined mitochondrial D-loop methylation in SMCs from vascular stenotic-occlusive diseases. MSP primers for mouse tissues locates at 15,835 to 15,996 of the mouse mtDNA (Fig. 5a). Complete ligation of the left common carotid arteries was used to block blood flow to induce intimal hyperplasia (Fig. 5b). Both sides of carotid arteries were harvested. In the de-endothelialized intima-media layer, SMC was found the major cell type as indicated by SMC marker SMα-actin (Fig. S10). Compared with the unligated right carotid arteries, the left carotid arteries showed higher level of D-loop methylation (Fig. 5c), suggesting a correlation between intimal hyperplasia and D-loop methylation. We also employed another mouse model, in which intimal hyperplasia was induced by removal of endothelium in guide-wire-injured left carotid arteries (Fig. 5d). At 4 weeks post-surgery, increased D-loop methylation was observed in the intima-media SMCs from injured arteries compared with the uninjured side (Fig. 5e), which was similar to the ligation model.

To elucidate the clinical relevance of this study, we measured mitochondrial D-loop methylation in endarterectomy specimens from patients with carotid occlusive diseases. In some measurements the non-diseased internal mammary arteries from patients underwent artery bypass grafting served as controls. Immunofluorescent staining indicated the presence of SMCs in both the diseased and control arteries (Fig. S11A). Compared with the control arteries with a methylation ratio of 54% in average, the ratio in diseased arteries increased to about 70% (Fig. 5f). In consideration of the intrinsic difference between internal mammary artery and carotid arteries, we also compared the methylation levels of D-loop region in different areas within each carotid artery specimen, including three parts: distal area, proximal area and plaque area. Highest D-loop methylation level was found in the plaques area, whereas much lower methylation was found in the proximal area, and the lowest in the distal area (Fig. 5g). Immunofluorescent staining verified the presence of SMCs in all the three parts of the specimens (Fig. S11B). These findings provide strong evidence, suggesting that mitochondrial D-loop hypermethylation correlated with arterial stenotic-occlusive diseases.

### Mitochondrial gene expression, mitochondrial respiration, and vessel contraction are decreased in vascular stenotic-occlusive diseases

**Fig. 6:**
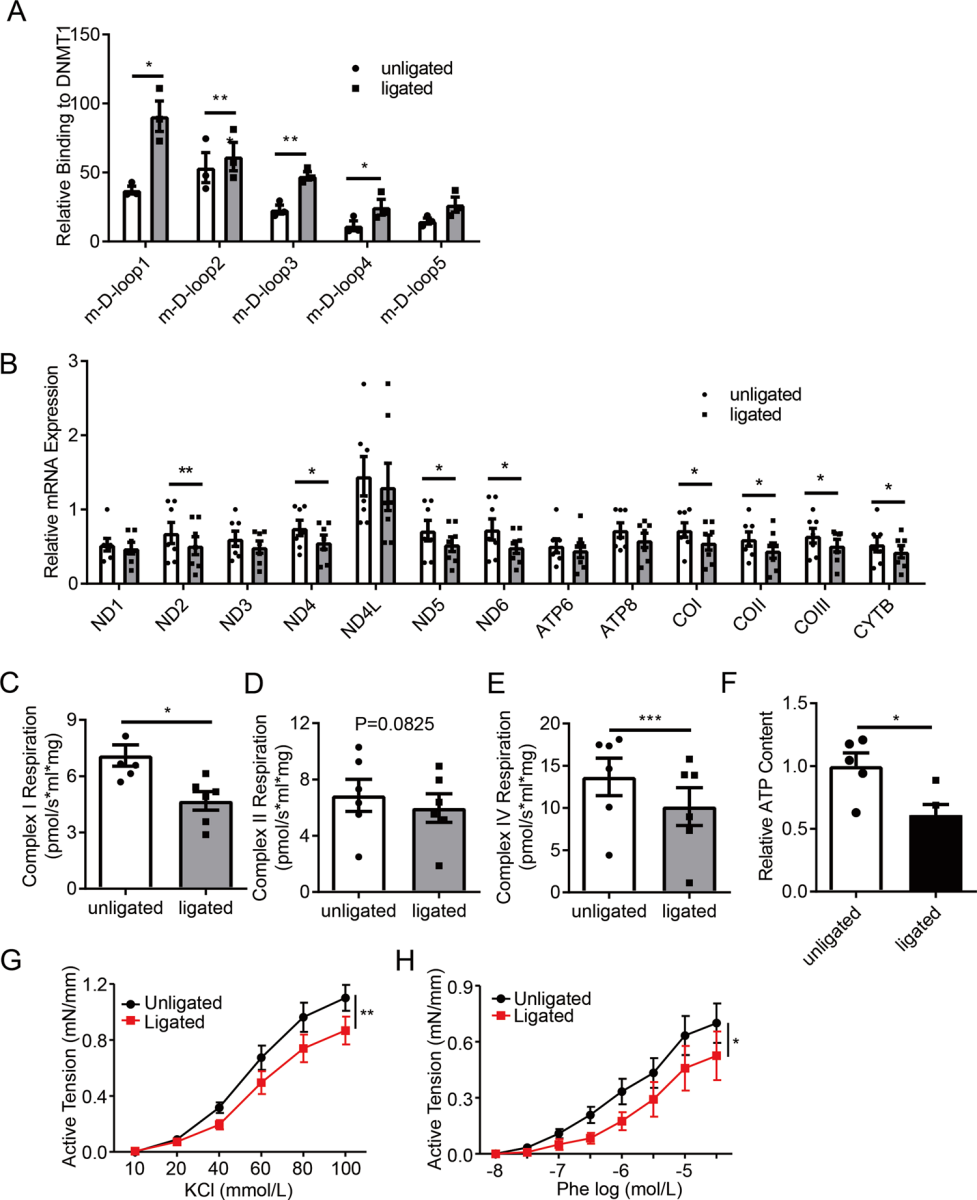
Vascular smooth muscle cells from stenotic arteries exhibit preferential cytoplasmic localization of DNMT1 and suppressed mitochondrial gene expression and function as well as contractility. Mouse carotid arteries on both sides were isolated 1 or 4 weeks after ligation. **a** DNMT1 binding to D-loop region was analyzed by chromatin immunoprecipitation (ChIP) assay (*n* = 3). **b** Transcriptional levels of 13 mtDNA genes were measured by quantitative RT-PCR (*n* = 7). **c**–**e** Complex I, II, and IV-supported respiration was analyzed by Oroboros Oxygraph-2k, a high-resolution respiration analyzer (*n* = 6). **f** ATP content was assessed by a luminometer *n* = 5. **g**, **h** Unligated or ligated carotid arteries were cut into ring segments of 3 mm in length and the ring segments were suspended in the myograph. KCl-simulated (*n* = 6) and phenylephrine-simulated (*n* = 4) vascular contraction were expressed as active tension (mN/mm). Phe phenylephrine. *P < 0.05, **P < 0.01, ***P < 0.005 by student’s *t*-test (**a**–**e**, **f**) and one-way ANOVA with Tukey’s post hoc analysis (**h**, **i**). Error bars show ± SEM.

ChIP assay to determine DNMT1 binding to the D-loop regions showed that four out of the five D-loop regions had increased association to DNMT1 in the ligated mouse carotid arteries (Figs. 5a and 6a). RT-PCR showed that several mtDNA-encoded genes, including ND2, ND4, ND5, ND6, COI, COII, COIII, and Cytb, were downregulated in the ligated versus the unligated arteries (Fig. 6b). Since mtDNA-encoded genes are components of the mitochondrial respiratory complexes I and IV, but not complex II^6^, we performed respirometry test using a mitochondrial high-resolution respiration system. The respiratory complex I and IV-supported respiratory function were markedly reduced in the ligated arteries, while the respiratory complex II-supported respiratory function had no significant change (Fig. 6c–e). ATP content in the de-endothelialized intima-media layer was also reduced in the ligated arteries (Fig. 6f), indicating a functional defect in ATP synthesis. Additionally, KCl- or phenylephrine- (Phe-) induced arterial ring contraction also decreased in the ligated arteries (Fig. 6g, h).

### Mitochondrial transplantation restores vascular contractility ex vivo

**Fig. 7:**
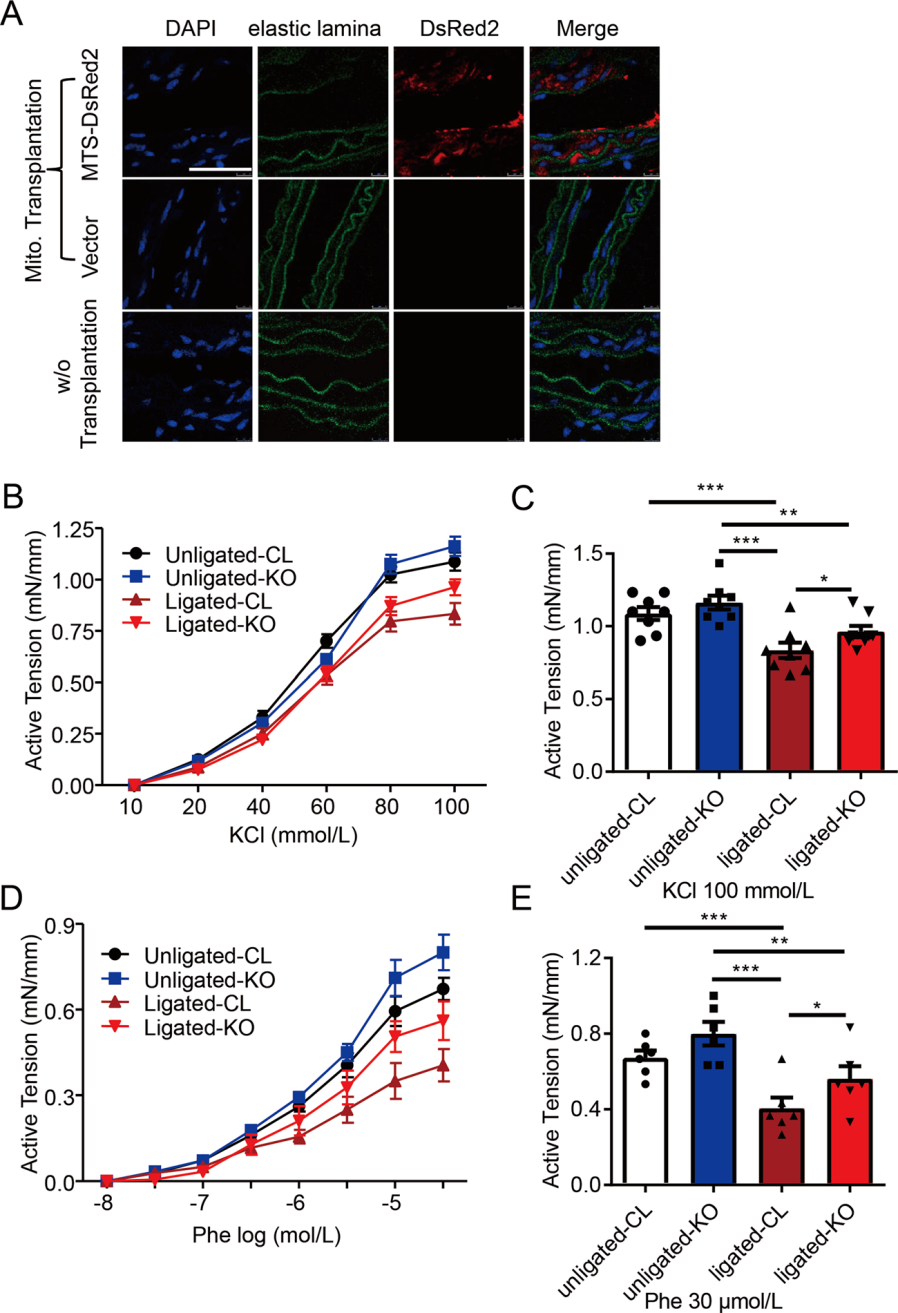
Mitochondrial transplantation restores vascular contractility ex vivo. **a** Immunocytochemical stainings of isolated common carotid artery segments in the absence (lower panels) or presence of mitochondrial suspensions (upper and middle panels). Mitochondrial suspensions were prepared from mouse embryonic fibroblasts (MEFs) transfected with MTS-DsRed2 plasmids (upper) or empty vectors (middle). Scale bar: 40 μm. **b**, **d** Unligated or ligated carotid arteries from C57/BL6 wild-type mice were cut into ring segments of 3 mm in length and the ring segments were incubated for 12 h with mitochondrial suspensions. The donor mitochondria were prepared from Dnmt1flox/flox MEFs with infection with control adenovirus (CL) or adenovirus expressing Cre recombinase protein (KO). KCl-simulated and phenylephrine-simulated vascular contraction were expressed as active tension (mN/mm). **c**, **e** Quantification of vascular contraction effect at 100 mM KCl (*n* = 8) and 30 μmol/L phenylephrine (*n* = 6) treatments in **b**, **d**. Phe, phenylephrine; KO, knockout. *P < 0.05, **P < 0.01, ***P < 0.005 by one-way ANOVA with Tukey’s post hoc analysis (**c**, **e**). Error bars show ± SEM.

In order to verify the role of mitochondrial DNMT1 in inhibiting vascular contractility, we investigated whether restoration of normal D-loop methylation level by transplanting mitochondria with Dnmt1 deletion rescues contractile function of ligated arteries ex vivo. Dnmt1 knockout was achieved by adenoviral expressing Cre mediated deletion of Dnmt1 allele in the mouse embryonic fibroblasts (MEFs) from Dnmt1^flox/flox^ mice (Fig. S12). Mitochondria were then isolated from the knockout (KO, with Cre expression) and control MEFs (Dnmt1^flox/flox^). The ligated and unligated arteries were dissected after 7 days post-ligation and then incubated with the isolated MEF mitochondria for 12 h. Entry of foreign mitochondria into the recipient vessels were detected by the fluorescence labeled donor mitochondria (Fig. 7a). Functional study showed that the KCl- or Phe- induced contraction did not significantly differ between unligated vessels receiving either control or Dnmt1 KO (Fig. 7b–e). Importantly, the impaired KCl- and Phe-induced contraction of the recipient ligated vessels was rescued after receiving Dnmt1 KO mitochondria (Fig. 7b–e). These data suggest that mitochondrial transplantation with Dnmt1 KO mitochondria restores vascular contractility impaired during vascular restenosis.

## Discussion

In this study, we provide the first evidence that, in vascular SMCs, DNMT1 translocate to the mitochondria in response to the pro-proliferative stimulus, and induce mtDNA hypermethylation. As a consequence, mtDNA transcription is repressed, causing mitochondrial dysfunction and reduction in ATP production, impairing SMC contractility in the context of vascular restenosis or occlusion.

Previous studies on DNMTs mainly focus on their regulation of nuclear DNA. The role of DNMT1 in regulating mitochondrial gene expression and function was largely unknown and overlooked possibly because methylation of mitochondrial genome was generally considered as nonexistent in early reports^26,27^. Recent years, accumulating evidence showed the presence of DNMT1 and DNMT3A inside mitochondria in support of the occurrence of mtDNA methylation^15,28,29^. DNMT1 was found to localize to mitochondria and cause mtDNA methylation, which affects mitochondrial biology in human lung cancer and colorectal carcinoma cell lines^16^. DNMT3A was also found in mitochondria of skeletal muscle and central nervous system from adult mouse or human^17^. Although contradictory reports also revealed an absence of CpG methylation in mitochondria, which might be due to different experimental conditions^12^, evidence favoring methylation of mtDNA methylation continued to emerge^30,31^. Here, we demonstrated not only the presence of DNMT1 and 5-methylcytosine in mtDNA, but also the translocation of DNMT1 from the nuclei to the mitochondria in response to PDGF-BB in vascular SMCs (Fig. 1). Our findings, along with the results of previous studies, suggest that cytosine methylation in mitochondrial genome is likely a common epigenetic modification in various cell types. Furthermore, our data showed that mitochondrial-specific targeting DNMT1 increased the methylation level of mitochondrial D-loop region and downregulated transcriptions of ND4, ND4L, ND5, ND6, ATP6, COI and COIII (Fig. 2c, d), raising the possibility that DNMT1 regulates mtDNA-encoded gene expression. Nevertheless, in addition to inducing mtDNA methylation, DNMT1 might also directly interfere with mtDNA transcription via interacting with the transcription machinery to suppress initiation of transcription, which needs future exploration.

The mtDNA-encoded 13 polypeptides are components of the oxidative phosphorylation system and are directly involved in cellular respiration, generating the majority of ATP required for cell metabolism^6,32^. Abnormally elevated expressions of mitochondrial gene have been linked to various pathological conditions^33,34^. On the other side, deficient mitochondrial gene expression and the subsequent impairment in oxidative phosphorylation leads to cellular functional defect. For example, disruption of the mitochondrial transcription factor A (Tfam) gene resulted in severe respiratory chain deficiency and increased susceptibility to apoptosis in the heart^35^. SMC-specific abrogation of Tfam in mice led to a reduction in arterial contraction in response to phenylephrine^8^. However, there is no study using direct manipulation of the mtDNA gene due to the limitation of the current experimental techniques. Our study provides a direct proof showing that suppression of mitochondrial gene expression by mitochondria-specific DNMT1-mediated hypermethylation causes mitochondrial dysfunction such as ROS and ATP production, mtDNA replication, and mitochondrial respiration in both cells and isolated vessels (Fig. 2e–j).

Depicting the mitochondrial structure, assembly, coupling mechanism and pathology of respiratory chain complexes has been drawing much attention for mitochondrial biologists^36^. The mitochondrial electron transfer chain consists of five distinct complexes^37^, among which the complexes I–IV function as proton pumps to translocate protons from the mitochondrial matrix into the intermembrane space. The electron transport generates a proton gradient across the mitochondrial inner membrane, which drives synthesis of ATP via complex V. Our data showed that in ligation-induced stenotic arteries, the activity of complex I and IV was inhibited (Fig. 6c, e), with the downregulation of ND2, -4, -5, and -6 (components of complex I) and COI-III (component of complex IV) encoded by mtDNA (Fig. 6b). These mtDNA-encoded subunits play an important role in proton pumping from the matrix and are therefore critical for mitochondrial respiration^38^. In line with these result, in cultured SMCs, mitochondrial-targeting DNMT1 reduced ROS production (Fig. 2e), probably due to the suppression of complex I as an important site for generating ROS. Thus, the mitochondrial DNMT1 resulted decrease in ROS production might lead to deficiency in cellular responses to stresses.

Vascular SMCs are highly plastic and capable of phenotypic switch, depending on the environmental cues they sense^39^. Epigenetic modification is one of the regulatory mechanisms of SMC phenotype^40^. For example, DNA methylation modulates the expression of SMC phenotypic markers, such as SM22α and alkaline phosphatase^41,42^. Our previous study also showed that in vascular SMCs, DNMT1 is important for the matrix stiffness-induced phenotypic switch and its deficiency causes arterial stiffening, likely attributable to nuclear DNMT1^43^. In the current study, we revealed a different role of mitochondrial DNMT1 in regulating the contractile phenotype of vascular SMC, extending our understanding of the role of DNA methylation in directing SMC phenotype through regulating mitochondrial bioenergetics-related mtDNA-encoded gene transcription (Figs. 3 and 4, and Fig S7), also suggesting that maintaining mitochondrial respiration might be beneficial on cell contractility and could be target for treating vascular diseases.

Studies have shown that mtDNA methylation status changes in human diseases, such as type 2 diabetes^44^, polycystic follicle syndrome^45^, Alzheimer’s disease and Parkinson’s disease^46^. These studies illustrated a potentially important role of mtDNA methylation in disease development. However, they were usually limited to demonstrating the correlation between mtDNA methylation and disease. The definitive role of mtDNA methylation in affecting cellular function has been little studied. To the best of our knowledge, there has been little evidence linking alterations in mtDNA methylation to vascular SMC function and the relative cardiovascular diseases. The major novel finding of our study is that inhibition of SMC contractility could be achieved by mitochondrial expression/localization of DNMT1 (Figs. 3 and 4). Using carotid complete ligation model and carotid guide-wire-injured model as well as human endarterectomy specimens, we demonstrated that hypermethylation status of mitochondrial D-loop region occurred in the diseased arteries (Fig. 5). Our results filled a research gap between mtDNA methylation and SMC function and may provide a molecular basis for further understanding the mechanisms of vascular homeostasis and dysfunction. Importantly, the mouse disease model combined with mitochondrial transplantation ex vivo (Fig. 7), showed the causal relationship between D-loop hypermethylation of mtDNA in SMCs and functional alterations in vascular stenotic-occlusive diseases could be well demonstrated. These evidences also indicated the potential therapeutic value of modulating mitochondrial bioenergetics to treat vascular disease.

## Supplementary information

Supplemental Figure 1

Supplemental Figure 2

Supplemental Figure 3

Supplemental Figure 4

Supplemental Figure 5

Supplemental Figure 6

Supplemental Figure 7

Supplemental Figure 8

Supplemental Figure 9

Supplemental Figure 10

Supplemental Figure 11

Supplemental Figure 12

Supplementary Table

Supplementary Figure Legends

Supplementary Material

CDDIS

DECLARATION OF CONTRIBUTIONS TO ARTICLE

Entire Unedited Gel
