# Supplementary figures and images for: Hypermethylation of mitochondrial DNA in vascular smooth muscle cells impairs cell contractility
Source: Cell Death Dis. 2020 Jan 20;11(1):35. doi: 10.1038/s41419-020-2240-7 (PMC6971246; doi:10.1038/s41419-020-2240-7)

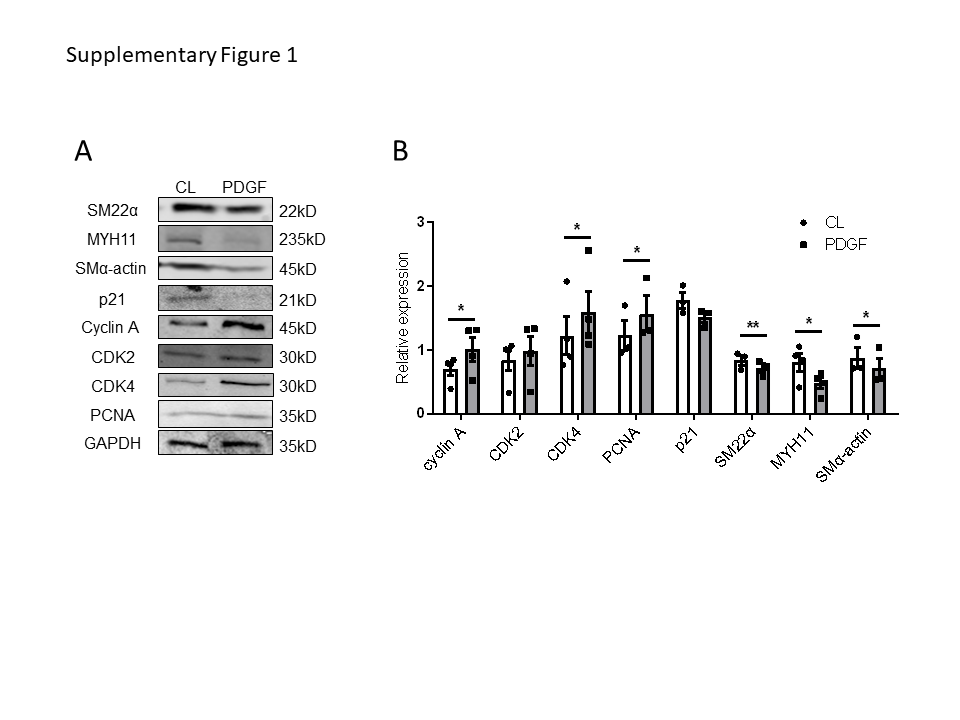

Supplement: Supplementary file 1 — Supplemental Figure 1 [file 41419_2020_2240_MOESM1_ESM.tif]

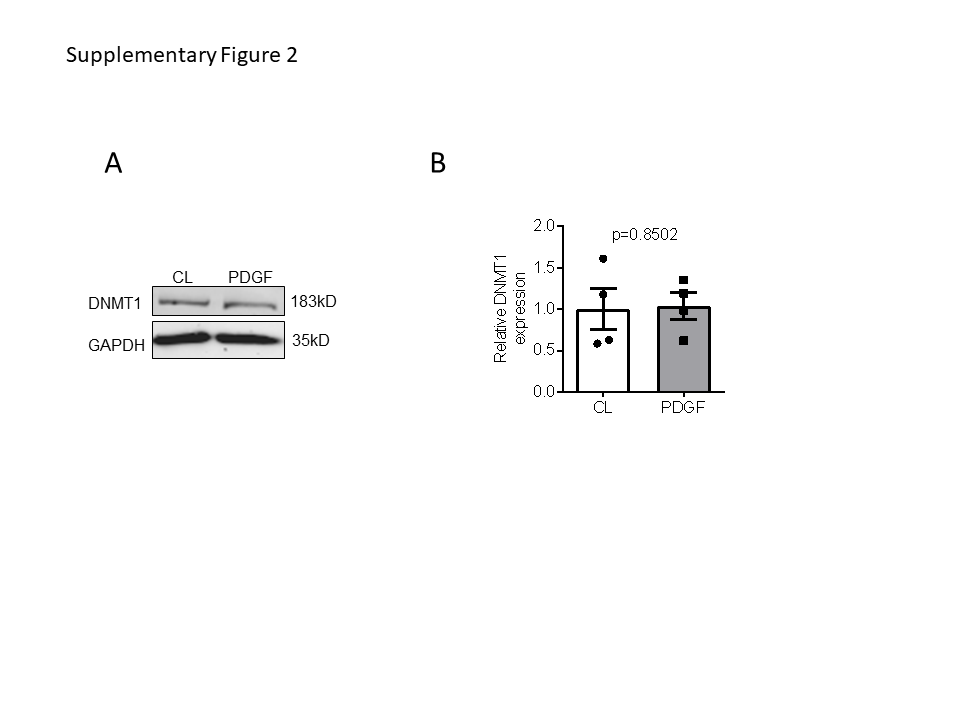

Supplement: Supplementary file 2 — Supplemental Figure 2 [file 41419_2020_2240_MOESM2_ESM.tif]

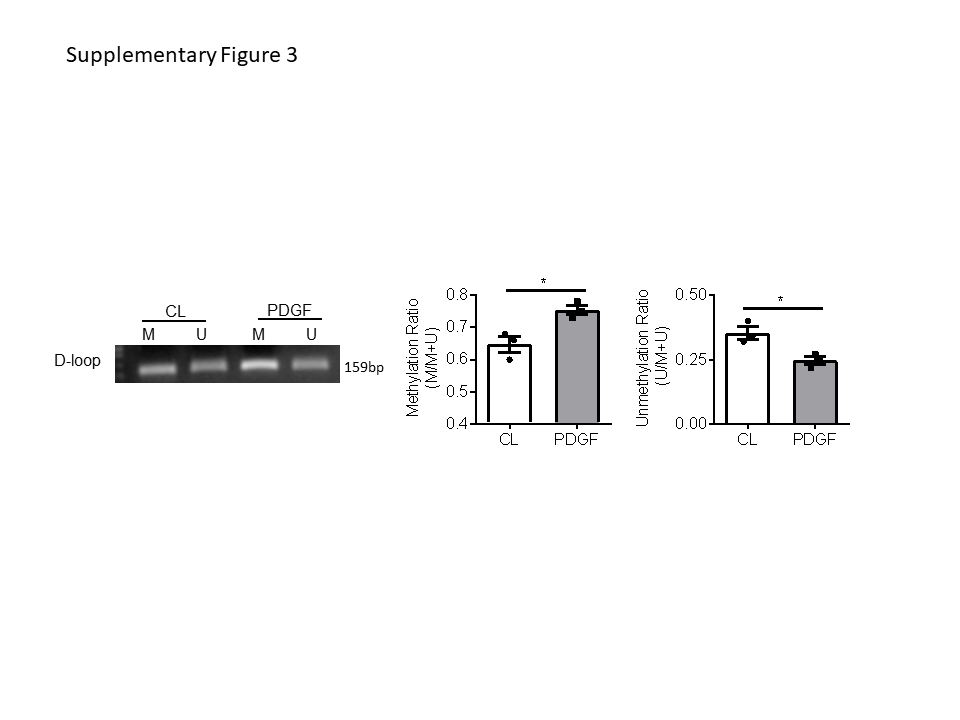

Supplement: Supplementary file 3 — Supplemental Figure 3 [file 41419_2020_2240_MOESM3_ESM.tif]

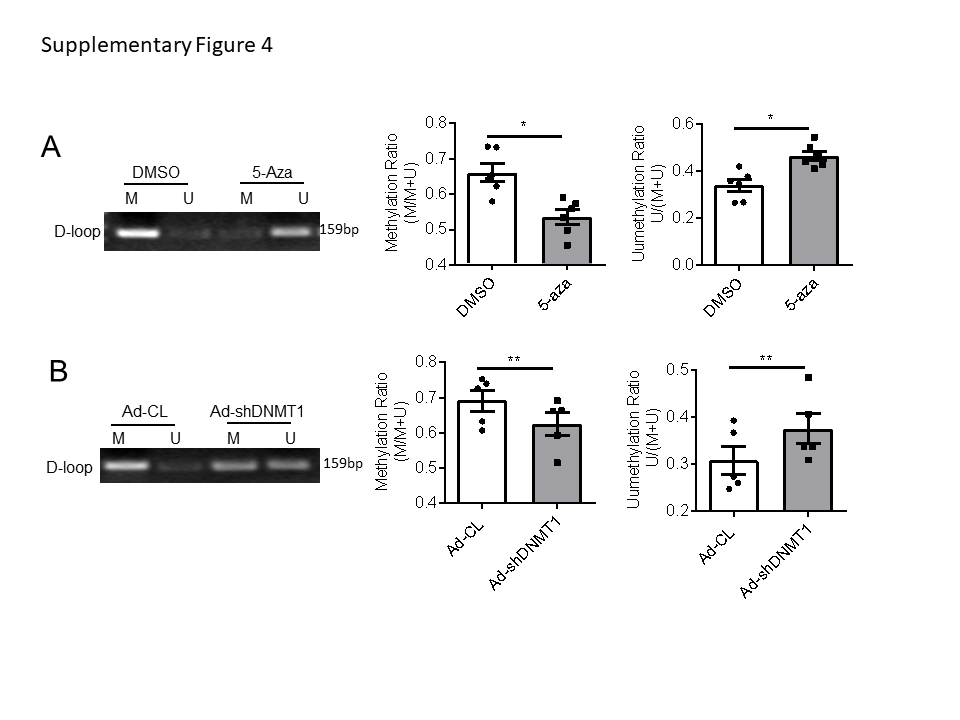

Supplement: Supplementary file 4 — Supplemental Figure 4 [file 41419_2020_2240_MOESM4_ESM.tif]

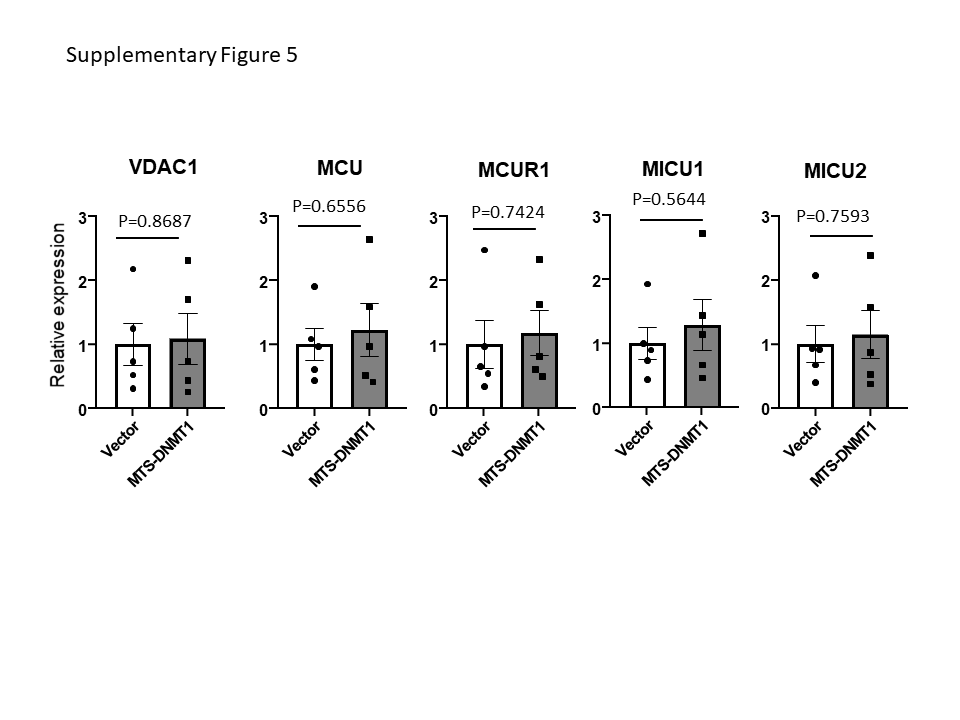

Supplement: Supplementary file 5 — Supplemental Figure 5 [file 41419_2020_2240_MOESM5_ESM.tif]

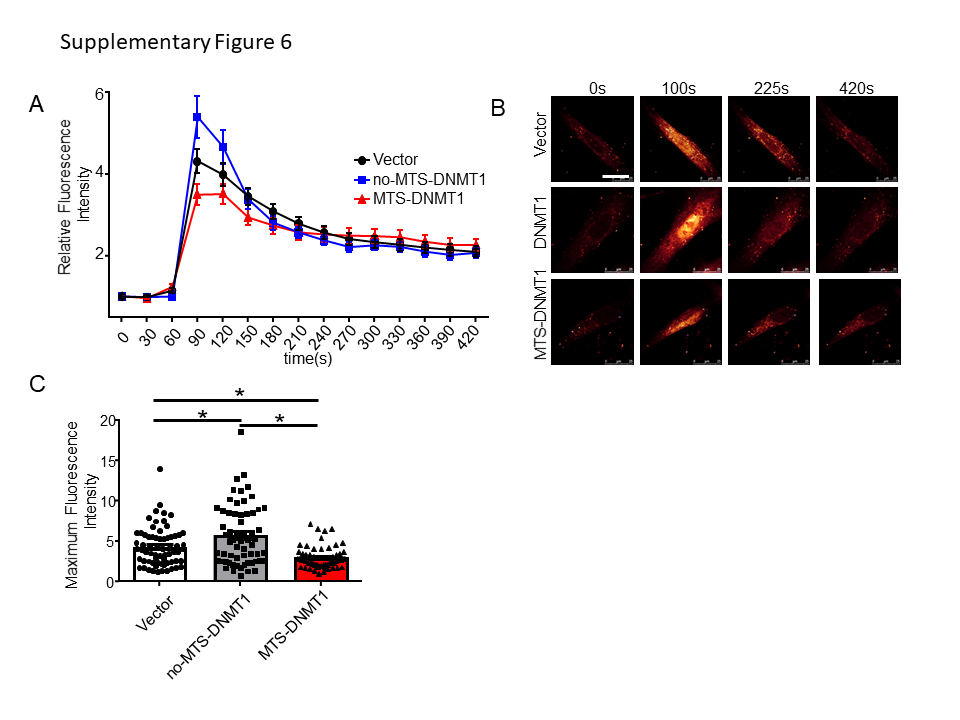

Supplement: Supplementary file 6 — Supplemental Figure 6 [file 41419_2020_2240_MOESM6_ESM.tif]

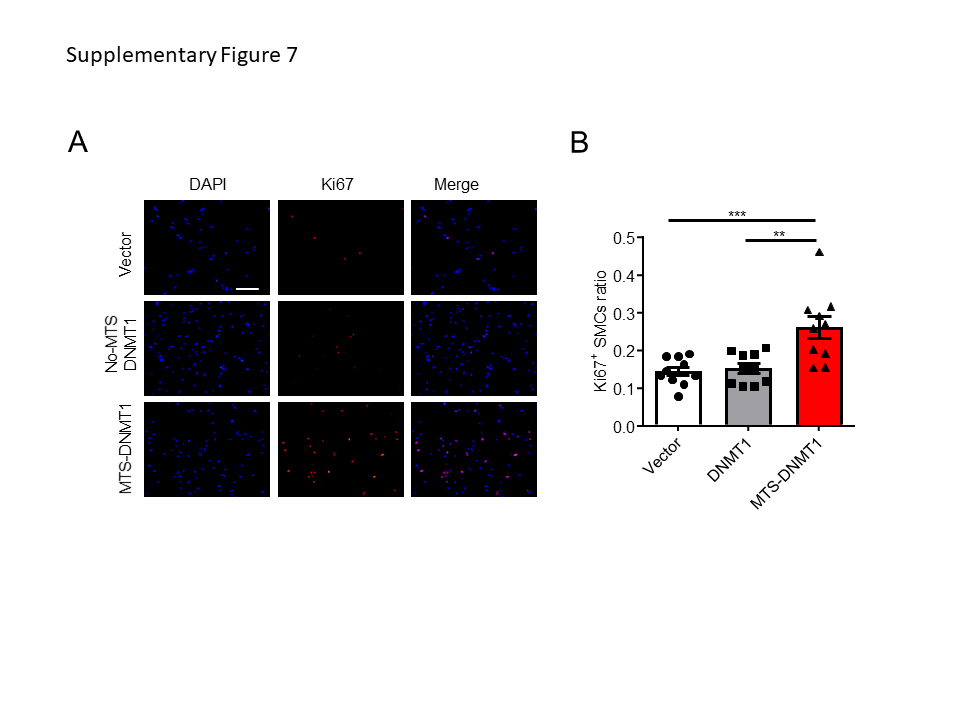

Supplement: Supplementary file 7 — Supplemental Figure 7 [file 41419_2020_2240_MOESM7_ESM.tif]

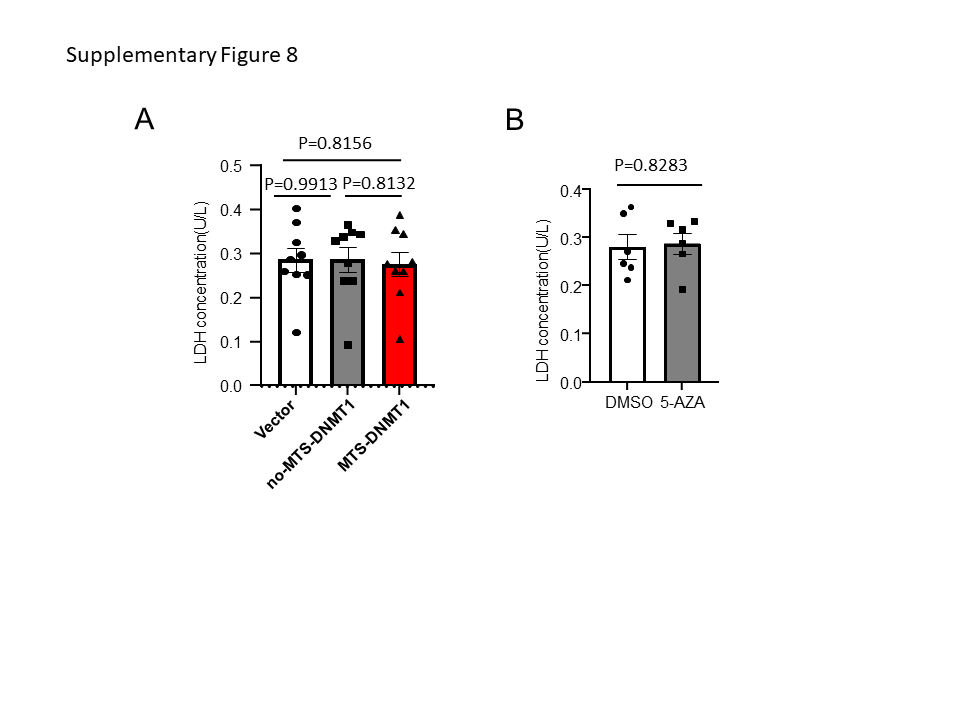

Supplement: Supplementary file 8 — Supplemental Figure 8 [file 41419_2020_2240_MOESM8_ESM.tif]

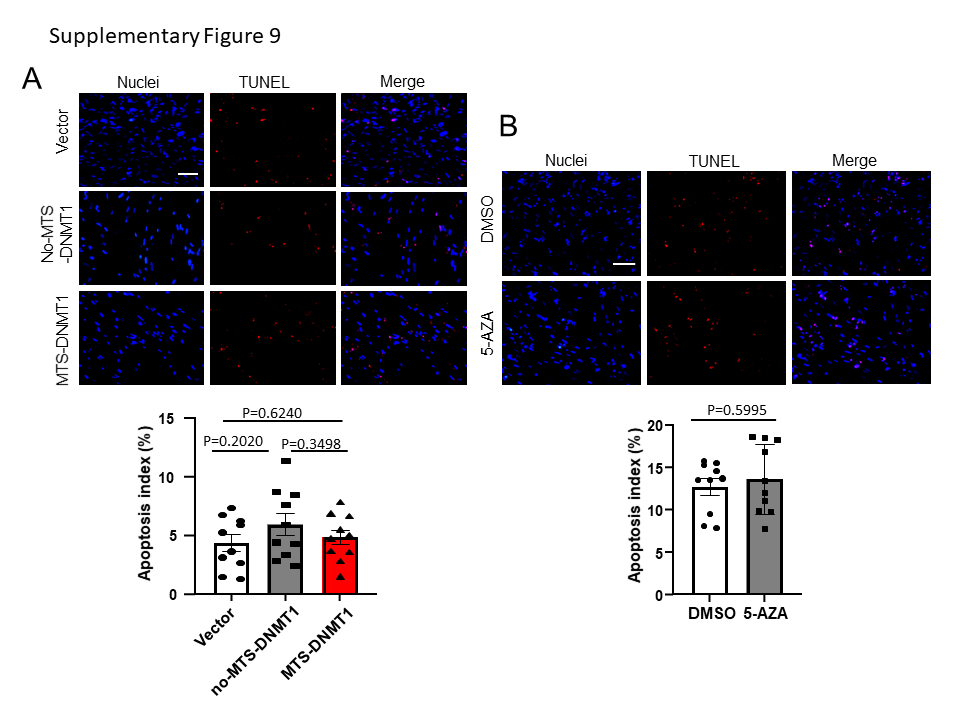

Supplement: Supplementary file 9 — Supplemental Figure 9 [file 41419_2020_2240_MOESM9_ESM.tif]

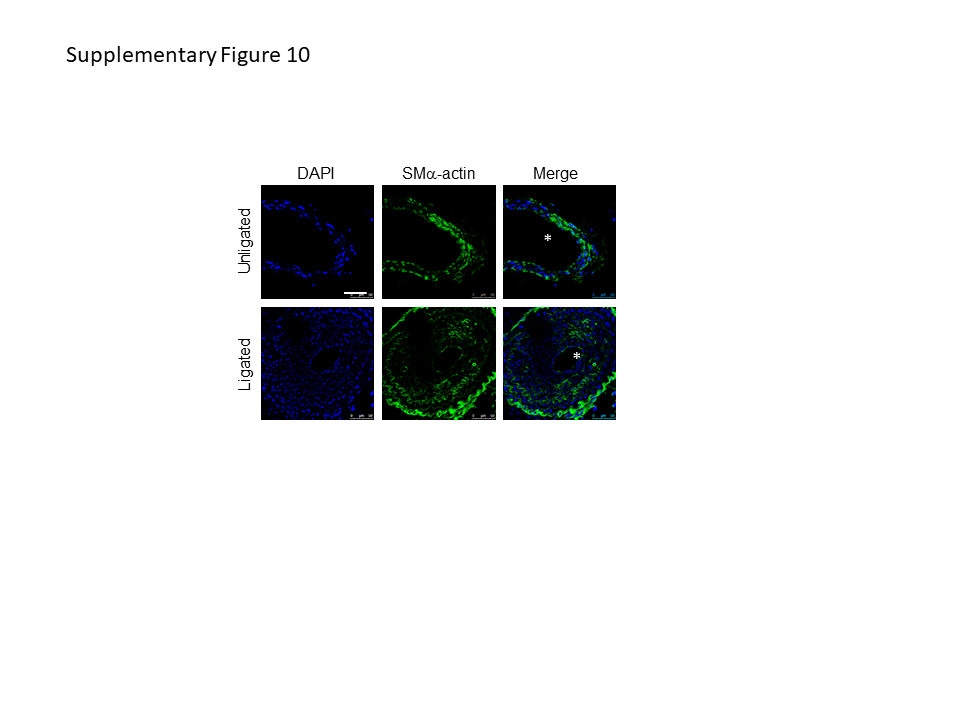

Supplement: Supplementary file 10 — Supplemental Figure 10 [file 41419_2020_2240_MOESM10_ESM.tif]

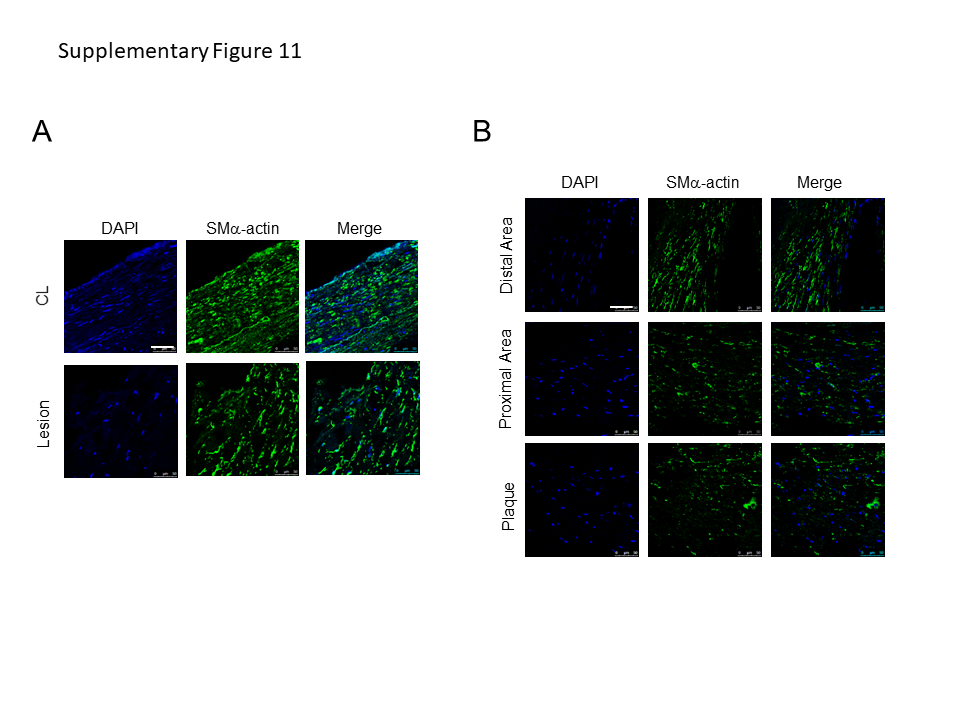

Supplement: Supplementary file 11 — Supplemental Figure 11 [file 41419_2020_2240_MOESM11_ESM.tif]

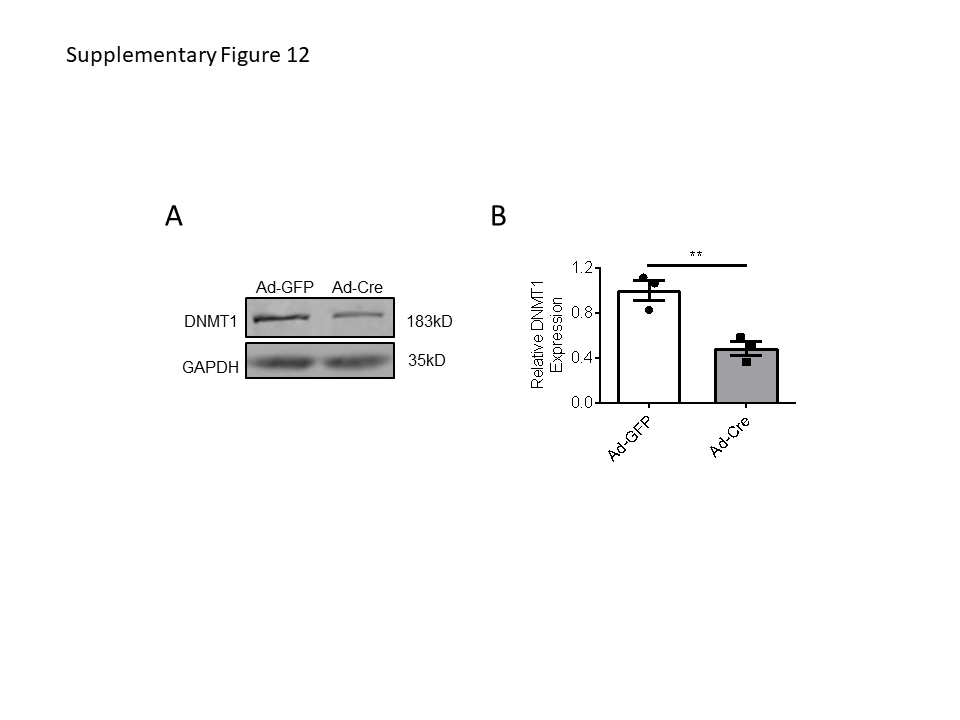

Supplement: Supplementary file 12 — Supplemental Figure 12 [file 41419_2020_2240_MOESM12_ESM.tif]
