# Supplementary material for: Hypermethylation of mitochondrial DNA in vascular smooth muscle cells impairs cell contractility
Source: Cell Death Dis. 2020 Jan 20;11(1):35. doi: 10.1038/s41419-020-2240-7 (PMC6971246; doi:10.1038/s41419-020-2240-7)
Supplement: Supplementary file 13 — Supplementary Table [file 41419_2020_2240_MOESM13_ESM.docx]

Table 1 Primer sets for PCR

| Primer | Sequence |
| --- | --- |
| h-chr5  h-D-loop 1  h-D-loop 2  h-D-loop 3  h-D-loop 4  h-D-loop 5  h-D-loop 6  m-D-loop 1  m-D-loop 2  m-D-loop 3 | 5’-CAGTAAGCCGTTCACTCTCACA-3’  5’-CTGTACCTGGGGTTCATTCATT-3’  5’-ATGGGGAAGCAGATTTGGGT-3’  5’-GAGGGTTGATTGCTGTACTTGC-3’  5’-ACTATCACACATCAACTGCAACT-3’  5’-TCACGGAGGATGGTGGTCAA-3’  5’-ATATCCCGCACAAGAGTGCT-3’  5’-CCCGTGAGTGGTTAATAGGGT-3’  5’-CGGGAGCTCTCCATGCATTT-3’  5’-ACACTTTAGTAAGTATGTTCGCCTG-3’  5’-ACAGGCGAACATACTTACTAAAGTG-3’  5’-AGGGTTCTTTGTTTTTGGGGT-3’  5’-CACCAGCCTAACCAGATTTCA-3’  5’-TTGCTTTGAGGAGGTAAGCTACA-3’  5’-CCCCAAGCATATAAGCTAGTACA-3’  5’-ATGACTGTATGGTGTATGTCAGAT-3’  5’-TCTCTTCCATATGACTATCCCCT-3’  5’-GGGGAACGTATGGGCGATAA-3’  5’-ttccccttaaataagacatctcgat-3’  5’-CACCGTAGGTGCGTCTAGACTGTGT-3’ |

| Primer | Sequence |
| --- | --- |
| m-D-loop 4  m-D-loop 5  m-ND1  m-ND2  m-ND3  m-ND4  m-ND4L  m-ND5  m-ND6  m-ATP6 | 5’-GACGCACCTACGGTGAAGAA-3’  5’-CTTTCAAGTTCTTAGTGTTTTTGGG-3’  5’-GTCCTGATCAATTCTAGTAGTTCCC-3’  5’-AGAGTTTTGGTTCACGGAACAT-3’  5’-TGCCTGCCCAGTGACTAAAG-3’  5’-GACCCTCGTTTAGCCGTTCA-3’  5’-ATCCTCCTGGCCATCGTACT-3’  5’-ATCAGAAGTGGAATGGGGCG-3’  5’-CTAGTTGCATTCTGACTCCCCC-3’  5’-AGAATGGTAGACGTGCAGAGC-3’  5’-ACCCGATGAGGGAACCAAAC-3’  5’-AGCGTCTAAGGTGTGTGTTGT-3’  5’-TCCACATTACTATGCCTGGAAGG-3’  5’-TAGTCCTACAGCTGCTTCGC-3’  5’-TAACCGCATCGGAGACATCG-3’  5’-GTGGAGGCCAAATTGTGCTG-3’  5’-TATTGCCGCTACCCCAATCC-3’  5’-TCCAGAGACTTGGGGATCTAACT-3’  5’-GCAGTCCGGCTTACAGCTAA-3’  5’-GGTAGCTGTTGGTGGGCTAA-3’ |

| Primer | Sequence |
| --- | --- |
| m-ATP8 | 5’-ACAAACATTCCCACTGGCAC-3’  5’-TTGGGGTAATGAATGAGGCAA-3’ |
| m-COI  m-COII  m-COIII  m-Cytb  h-ND1  h-ND2  h-ND3  h-ND4  h-ND4L | 5’-TCGGAGCCCCAGATATAGCA-3’  5’-TTTCCGGCTAGAGGTGGGTA-3’  5’-TAACCGAGTCGTTCTGCCAA-3’  5’-ACCCTGGTCGGTTTGATGTT-3’  5’-CTACCAAGGCCACCACACTC-3’  5’-AGGTCAGCAGCCTCCTAGAT-3’  5’-ACCTCAAAGCAACGAAGCCT-3’  5’-TACTGGTTGGCCCCCAATTC-3’  5’-CGATTCCGCTACGACCAACT-3’  5’-AGGTTTGAGGGGGAATGCTG-3’  5’-ACCAAACCCAGCTACGCAAA-3’  5’-AGTAGTAGGGTCGTGGTGCT-3’  5’-GCGGCTTCGACCCTATATCC-3’  5’-AGGGCTCATGGTAGGGGTAA-3’  5’-TCGCTCACACCTCATATCCTC-3’  5’-AGGCGGCAAAGACTAGTATGG-3’  5’-CAGCCACATAGCCCTCGTAG-3’  5’-CCCGTGGGCGATTATGAGAA-3’ |

| Primer | Sequence |
| --- | --- |
| h-ND5  h-ND6  h-ATP6  h-ATP8  h-COI  h-COII  h-COIII  h-Cytb  h-D-loop M  h-D-loop U | 5’-TCATCGCTACCTCCCTGACA-3’  5’-ATCCTGCGAATAGGCTTCCG-3’  5’-ACCTATTCCCCCGAGCAATC-3’  5’-GGGAGGATCCTATTGGTGCG-3’  5’-CGTACGCCTAACCGCTAACA-3’  5’-AGGCGACAGCGATTTCTAGG-3’  5’-TACCACCTACCTCCCTCACC-3’  5’-AGGATTGTGGGGGCAATGAAT-3’  5’-ACCCTAGACCAAACCTACGCCAAA-3’  5’-TAGGCCGAGAAAGTGTTGTGGGAA-3’  5’-CCGTCTGAACTATCCTGCCC-3’  5’-GAGGGATCGTTGACCTCGTC-3’  5’-CAGCCCATGACCCCTAACAG-3’  5’-TACATCGCGCCATCATTGGT-3’  5’-AGTCCCACCCTCACACGATTCTTT-3’  5’-AGTAAGCCGAGGGCGTCTTTGATT-3’  5’-CGTTTTTTTTAAATAAGATATTACGA-3’  5’-AAAAATCAAAAACAAATACTACGAC-3’  5’-TGTTTTTTTTAAATAAGATATTATGA-3’  5’-AAAAATCAAAAACAAATACTACAAC-3’ |
|  |  |

| Primer | Sequence |
| --- | --- |
| m-D-loopM  m-D-loop U  m-GAPDH  h-GAPDH  h-VDAC1  h-MCU  h-MCUR1  h-MICU1  h-MICU2 | 5’-GTTTTTTTTAAATAAGATATTTCGA-3’  5’-TACTATCCTTTCATACCTTAACGAC-3’  5’-GTTTTTTTTAAATAAGATATTTTGA-3’  5’-TACTATCCTTTCATACCTTAACAAC-3’  5’-GGACCTCATGGCCTACATGG-3’  5’-TAGGGCCTCTCTTGCTCAGT-3’  5'-AATGGGCAGCCGTTAGGAAA-3'  5'-GCCCAATACGACCAAATCAGAG-3  5’- CCCTTGGTGAAGACATCCCT -3’  5’- CTGGTAGTCTTAGTGCTAGGTG -3’  5’- CCAGAAGCCAGAGACAGACAAT -3’  5’- AAGAACCGTGGCTGTGGATA -3  5’- ACAGGCTGCCCTAGATTTCAC -3’  5’- TATGTGAGGGCCTTTCCCTG -3’  5’- TACACCCACAATTGCTCACAC -3’  5’- TTTGCAGTTGACTCTCCACC -3’  5’- ACGAAGAGACGGTTTCCCAA -3’  5’- AGATTGTCCTAGAAGTGACTGGG -3’ |

h: human; m: mouse; M: methylation primers; U: unmethylation primers
