# Supplementary material for: Hypermethylation of mitochondrial DNA in vascular smooth muscle cells impairs cell contractility
Source: Cell Death Dis. 2020 Jan 20;11(1):35. doi: 10.1038/s41419-020-2240-7 (PMC6971246; doi:10.1038/s41419-020-2240-7)
Supplement: Supplementary file 14 — Supplementary Figure Legends [file 41419_2020_2240_MOESM14_ESM.docx]

**Supplementary Figure Legends**

**Figure S1. Expression of vascular SMC contractile and proliferative markers in cells subjected to PGDF-BB versus control treatments.** Vascular SMCs were exposed to vehicle control(CL) or PDGF-BB(20ng/ml) for 24h, expressions of the contractile markers (SM22a, MYH11, and SMa-actin), cell cycle repressor (p21), cell cycle regulators (Cyclin A, CDK2, and CDK4), as well as proliferative marker gene PCNA, were analyzed using Western blot assay. **(A)** Representative bands. **(B)** Statistical graph (n=3-4). PDGF, PDGF-BB. *P< 0.05 ***P*< 0.01 by student’s t test. Error bars show±SEM.

**Figure S2. Expression of DNMT1 in cells subjected to PGDF-BB versus control treatments.** Vascular SMCs were exposed to vehicle control(CL) or PDGF-BB(20 ng/ml) for 24h, and expression of DNMT1 was analyzed using Western blot assay. **(A)** Representative bands. **(B)** Statistical graph (n=4). PDGF, PDGF-BB. Data was analyzed by student’s t test. Error bars show±SEM.

**Figure S3. Methylation level of the D-loop region in mitochondria is increased by PDGF-BB treatment.** DNAs were extracted from isolated SMC mitochondria. Methylation status of the D-loop region in mitochondria was measured by MSP (n=3). M: methylated; U: unmethylated. **P*< 0.05 by student’s t test. Error bars show±SEM.

**Figure S4. Methylation level of the D-loop region is reduced by inhibition of DNMT1. (A)** Vascular SMCs were treated with control reagents (DMSO) or DNMT1 inhibitor, 5-Aza-2'-deoxycytidine (5-Aza) for 3 days. The methylation and unmethylation levels of D-loop region were measured by Methylation specific PCR (MSP) (n=6). **(B)** Vascular SMCs were infected with control adenovirus (Ad-CL) or recombinant adenovirus expressing shRNA specifically targeting human DNMT1 (ad-shDNMT1). The methylation and unmethylation levels of D-loop region were measured by MSP (n=5). M: methylated; U: unmethylated. **P*< 0.05 ***P*< 0.01 by student’s t test. Error bars show±SEM.

**Figure S5. Expressions of a series of nuclear DNA-encoded mitochondrial functional genes in cells expressing the no-MTS-DNMT1.** SMCs were transfected with no-MTS-DNMT1 and the control vectors. Gene expression was measured by quantitative RT-PCR assay. Error bars show±SEM.

**Figure S6. Expression of mitochondrial-targeting DNMT1 impairs smooth muscle cell Ca^2+^ influx. *A*,** Cells were transfected with empty vectors, no-MTS DNMT1, or MTS-DNMT1 and the real-time Ca^2+^ imaging was performed using fluorescence confocal microscopy. Data were obtained from triplicate biological replicates. ***B*,** Representative real-time Ca^2+^ images of single cell at different time points. Scale bar: 25μm.  ***C*,** Maximum fluorescence intensity was calculated from ***A***. **P*< 0.05 by 1-way ANOVA with Tukey’s post hoc analysis. Error bars show±SEM.

**Figure S7. Expression of exogenous mitochondrial-targeting DNMT1 promote cell proliferation.** Vascular SMCs were transfected with vector, no-MTS-DNMT1, or MTS-DNMT1 plasmids. Cell proliferation was determined by immunofluorescent staining of Ki67. **(A)** Representative images. Scale bar: 50μm. **(B)** Statistical graph. Results are mean±SEM from triplicate experiments with 10 randomly selected microscopic fields of each experiment. ***P*< 0.01 ****P*< 0.005 by 1-way ANOVA with Tukey’s post hoc analysis. Error bars show±SEM.

**Figure S8. Overexpression or inhibition of DNMT1 does not cause SMC necrosis.** Vascular SMCs were either transfected with vector, no-MTS-DNMT1, or MTS-DNMT1 plasmids **(A)**, or treated with 5-Aza-2'-deoxycytidine (a DNMT1 inhibitor) **(B)**. Necrosis was determined by lactate dehydrogenase (LDH) release assay. By student’s t test or1-way ANOVA with Tukey’s post hoc analysis. Error bars show±SEM.

**Figure S9. Overexpression or inhibition of DNMT1 does not cause SMC apoptosis.** Vascular SMCs were either transfected with vector, no-MTS-DNMT1, or MTS-DNMT1 plasmids **(A)**, or treated with 5-Aza-2'-deoxycytidine (a DNMT1 inhibitor) Scale bar: 100μm. **(B)**. Apoptosis was determined by TUNEL staining. Scale bar: 100μm. By student’s t test or1-way ANOVA with Tukey’s post hoc analysis. Error bars show±SEM.

**Figure S10. Presence of SMa-actin-positive cells in the ligated and unligated carotid arteries.** Frozen sections of unligated and ligated vessels were subjected to immunofluorescent staining. Shown are representative images. Scale bar: 50μm. * lumen

**FigureS11. Presence of SMa-actin-positive cells in human specimens. (A)** Representative immunofluorescent images of human internal mammary arteries (CL) and endarterectomy specimens from patients with carotid occlusive diseases (Lesion). SMa-actin indicated SMCs in these specimens. Scale bar: 50μm. **(B)** Representative immunofluorescent images of different parts in endarterectomy specimens from patients with carotid occlusive diseases. SMa-actin indicated SMCs in these specimens. Scale bar: 50μm.

**Figure S12. DNMT1 knockout in mouse embryonic fibroblasts (MEFs).** MEFs isolated from Dnmt1^flox/flox^ mice and were exposed to control adenovirus (Ad-GFP) or adenovirus expressing the Cre recombinase (Ad-Cre) for 3 days, and expression of Dnmt1 was analyzed using western blot assay. **(A)** Representative bands. **(B)** Statistical graph (n=3). ***P*< 0.01 by student’s t test. Error bars show±SEM.
