# Supplementary material for: Hypermethylation of mitochondrial DNA in vascular smooth muscle cells impairs cell contractility
Source: Cell Death Dis. 2020 Jan 20;11(1):35. doi: 10.1038/s41419-020-2240-7 (PMC6971246; doi:10.1038/s41419-020-2240-7)
Supplement: Supplementary file 15 — Supplementary Material [file 41419_2020_2240_MOESM15_ESM.pdf]

Supplemental Materials and Methods

Cell culture

Primary human umbilical artery smooth muscle cells (SMCs) were isolated from human umbilical artery and SMCs within passages 4 to 8 were used for all experiments *in vitro*. SMCs were maintained in Nutrient Mixture F12 Ham Kaighn's Modification (F12K, Sigma Aldrich) supplemented with 20% (for cell maintaining) or 2% (for treatment) fetal bovine serum (FBS) (Gemini) and 10% SMC Growth Medium (Cell Applications). Mouse embryonic fibroblasts (MEFs) were isolated from $Dnmt1^{flox/flox}$ mice embryo and were maintained in dulbecco's modified eagle medium (DMEM) (Gibco) supplemented with 10% fetal bovine serum (FBS) (Gemini).

Mitochondrial fractionation

Cultured SMCs were treated with PDGF-BB (20 ng/ml) or control reagent for 24 hours, and then digested by 0.05% trypsin and resuspended in Mito-isolate buffer (20 mmol/L HEPES, 20 mmol/L KCl, 1.5 mmol/L $MgCl_2$, 250 mmol/L sucrose, 1 mmol/L EDTA, 1 mmol/L PMSF, 1 mmol/L dithiothreitol, PH 7.5). The swelling cells were ruptured by glass homogenizer and then were centrifuged at 700g for 10 minutes, and the sediment was preserved as nucleus. The supernatant was centrifuged at 7000g for 10 minutes. The mitochondria sediment was washed by MS buffer (210 mmol/L mannitol, 70 mmol/L sucrose, 5 mmol/L Tris base, 5 mmol/L EDTA, PH 7.5). The nucleus and mitochondrial fraction were preserved for protein extraction. All experimentations were processed at 4°C.

Western blot assay

Cultured SMCs were lysed in the RIPA lysis buffer (25 mmol/L HEPES, 1% Triton X-100, 1% deoxycholate,

0.1% SDS, 125 mmol/L NaCl, 5 mmol/L EDTA, 50 mmol/L NaF, 1 mmol/L PMSF, pH 7.4). Protein concentrations were measured to ensure that equal amounts of protein were separated on SDS-PAGE and transferred to nitrocellulose membranes. Nonspecific binding was blocked in 5% skimmed milk in Tris-buffered saline (TBS) containing 0.1% Tween-20 (TBST). The membrane was then incubated with specific primary antibodies against DNMT1 (Santa Cruz Biotech), DNMT3A (ABclonal), DNMT3B (ABclonal), GAPDH (Santa Cruz Biotech), VDAC1 (Absin), Histone 3 (Beyotime), PCNA (Santa Cruz Biotech), CDK4 (Santa Cruz Biotech), CDK2 (Santa Cruz Biotech), p21 (Santa Cruz Biotech), Cyclin A (Santa Cruz Biotech), SMα-actin (Santa Cruz Biotech)，MYH11 (Santa Cruz Biotech)，calponin (Santa Cruz Biotech) or SM22α (Santa Cruz Biotech) overnight at 4°C, donkey anti-rabbit/-mouse/-goat IgG (H&L) was used as second antibodies (Rockland). Visualization was performed with an Odyssey infrared imaging system (LI-COR Biosciences).

Immunofluorescent staining of the cell

SMCs on glass slides with or without pre-incubation with mito-tracker (Invitrogen) were fixed in 4% paraformaldehyde (PFA) after washed by PBS, and then permeabilized with 0.25% Triton X-100 in PBS. Nonspecific binding was blocked by 3-5% bovine serum albumin (BSA) in PBS for 1 hour. The cells were probed with primary antibody against DNMT1 (Santa Cruz Biotech) overnight at 4°C. The cells were incubated with secondary antibodies including Alexa Fluor 488-conjugated donkey/goat anti-rabbit/-mouse IgG (1:500, ThermoFisher) or Alexa Fluor 555-conjugated donkey anti-rabbit/-goat IgG (1:500, ThermoFisher). Nuclei were counterstained with 4',6-diamidino-2-phenylindole (DAPI). The slips were visualized by fluorescence microscopy (Leica DMI6000B, Leica TCS SP8) or structured illumination microscopy (NIKON, A1 N-SIM STORM).

Chromatin immunoprecipitation (ChIP)

SMCs from culture or from media layer of blood vessels were washed with PBS, fixed with 1% formaldehyde in PBS for 10 minutes at room temperature, rinsed twice with ice-cold PBS, and scraped into 1 ml PBS at 4 °C. The pellet was resuspended with 200μl of lysis buffer (1% SDS, 5 mmol/L EDTA, 50 mmol/L Tris-HCl, protease inhibitor, pH 8.1) and incubated on ice for 10 minutes. The lysate was sonicated 12 times for 1.5 seconds each, and 1:1 diluted in dilution buffer (1% Triton X-100, 2 mmol/L EDTA, 150 mmol/L NaCl, 20 mmol/L Tris-HCl, protease inhibitor, pH 8.1), and incubated with IgG or anti-DNMT1 antibody overnight at 4 °C. Protein A-G sepharose beads (Santa Cruz Biotech) were added into the lysate and incubated with the lysate for another 2 hours at 4 °C. The beads-antibody-DNA complexes were washed sequentially with buffer TSE I (0.1% SDS, 1% Triton X-100, 2 mmol/L EDTA, 20 mmol/L Tris-HCl, 150 mmol/L NaCl, pH 8.1), buffer TSE II (0.1% SDS, 1% Triton X-100, 2 mmol/L EDTA, 20 mmol/L Tris-HCl, 500 mmol/L NaCl, pH 8.1), buffer III (0.25 mol/L LiCl, 1% NP-40, 1% deoxycholate, 1 mmol/L EDTA, 10 mmol/L Tris-HCl, pH 8.1), and TE buffer (10 mmol/L Tris-HCl,1 mmol/L EDTA, pH 8.0). Crosslinking were reversed at 65 °C for 6 hours or overnight. DNA was eluted with nuclease-free water after applied to DNA Pure-Spin Kit (Vigorous), and subjected for PCR amplification. DNMT1 bindings were quantified relative to a genomic intragenic region (Chr5) with a CpG dinucleotides percentage below 40%. Primer sets are listed in Table S1.

Methylation specific-polymerase chain reaction (MSP)

Genomic DNA was extracted for methylation analysis from cultured cells, blood vessels, and human specimens. One gram of genomic DNA was treated with sodium bisulfite by using EpiMark Bisulfite kit (BioLabs) according to the manufacturer's instructions. The converted DNA was eluted with nuclease-free water. PCR amplifications were performed for methylated (M) and unmethylated (U) regions in a total volume

of 50μL by using 2× EasyTaq PCR SuperMix (TRANSGEN BIOTECH). The reactions were subjected to initial incubation at 95 °C for 5 minutes, followed by 40-45 cycles of 95 °C for 30 seconds, and annealing at an appropriate temperature for 30 seconds and 72 °C for 30 seconds, final extension was done by incubation at 72 °C for 5 minutes. The MSP products were separated on 2% agarose gel and visualized after StarGreen DNA dye (DiNing) staining. The intensity of methylated bands or unmethylated bands to total intensity was quantified. Primer sets are listed in Table S1.

Viruses and plasmids

Ad-shDNMT1 carrying shRNA specifically targeting DNMT1 and the control adenovirus expressing GFP (Ad-GFP) were obtained from Vigene Biosciences. Adenovirus expressing the Cre recombinase (Ad-Cre) was obtained from SignaGen. pcDNA3/Myc-DNMT1 (no-MTS-DNMT1), in which the full-length cDNA for human DNMT1 was cloned into EcoRI and NotI sites of pcDNA3/Myc, was a gift from Arthur Riggs (Addgene plasmid # 36939). pDsRED2-Mito plasmid, in which the mitochondria targeting sequence (MTS) is fused to the 5'-end of pDsRed2, was from Clontech. The MTS-DsRED2 fragment was amplified by primer sets (forward primer: 5'-TCAGAGGAGGACCTGGAATTCATGTCCGTCCTGACGCCGC-3' reverse primer: 5'-ACCACCTGTTCCTGTAGGAATTCATGCCGGCGCGTACC-3') with the pDsRED2-Mito plasmid served as the template. The fragment was cloned into 939 sites of pcDNA3/Myc-DNMT1, upstream of DNMT1 open reading frame (ORF), to generate MTS-DNMT1. Validity of the MTS-DNMT1 construct was verified by base sequencing.

RNA isolation and quantitative RT-PCR

RNA was extracted from cultured cells or from vessels by TRIzol reagent (Life Technologies) according to the manufacturer's instructions. 1 mg of RNAs was reversed-transcribed into complementary DNA (cDNA) with

M-MLV RT system (Invitrogen) by using Oligo (dT) primers. Real-time PCR was performed with the 2X RealStar power SYBR Mixture (Genestar) by using specific primer pairs (Table S1). Gene expressions were normalized against GAPDH.

Measurement of reactive oxygen species (ROS) and mitochondrial membrane potential

Vascular SMCs were transfected with empty vectors, no-MTS DNMT1, or MTS-DNMT1. The ROS levels in cells were determined by using the DCFH-DA fluorescence indicator dye at a concentration of 10 μmol/L for 30 minutes in an incubator. Mitochondria membrane potential was determined by using the dye tetramethylrhodamine methyl ester (TMRM) at a concentration of 100 nmol/L for 30 minutes in an incubator. After incubation the cells were resuspended and washed by PBS and were then immediately monitored by flow cytometry.

Measurement of ATP content

Vascular SMCs were transfected with empty vectors, no-MTS DNMT1, or MTS-DNMT1, and the ATP content was measured using the ATP Detection Kit (Beyotime) according to the manufacturer's instructions. In brief, the cultured SMCs and media SMCs from vessels were resuspended in lysis buffer, and the supernatant was added into 96-well plate with ATP detection buffer. The RLU value was monitored by a luminometer and the ATP content was calculated using a standard curve.

DNA isolation and measurement of mitochondrial DNA copy number (mtDNAcn)

To extract DNA from cells or vessels, cells sediment or abrasive tissues were suspended in 50μl of TE buffer (10 mmol/L Tris-HCl,1 mmol/L EDTA pH 8.0), 450μl of STE buffer (150 mmol/L NaCl, 10 mmol/L Tris-HCl, 1 mmol/L EDTA, pH 8.0), 10μl of 20% SDS and 10μl of 20 mg/ml proteinase K at 55°C for 3 hours to overnight. Isopyknic Tris-phenol (Solarbio) was added into the suspension. The mixture was blended for 10

minutes at room temperature and centrifuged at 12000 rpm for 10 minutes. The water phase supernatant was removed to a new centrifuge tube. As mentioned above, the isopyknic Tris-phenol and DNA extraction buffer (phenol: trichloromethane: isoamylol=25:24:1, pH＞7.8) was added twice， and isopyknic trichloromethane and precipitation buffer (0.2 times the volume of 10 mol/L ammonium acetate, 10 times the volume of ethanol) was added once sequentially. 70% ethanol was added to wash the DNA sediment. 50μl-200μl of ddH_2O was added to dissolve and maintain DNA after the ethanol was evaporated.

Relative mtDNAcn in SMCs transfected with empty vectors, no-MTS DNMT1, or MTS-DNMT1 was measured by a quantitative reverse transcription PCR-based method. In brief, two pairs of primers were used in the two steps of relative quantification of mtDNAcn. One primer pair (5′-CCAACATCTCCGCATGATGAAAC-3′; 5′-TGAGTAGCCTCCTCAGATTC-3′) was used to amplify the mitochondrial CYTB gene, and the other primer pair (5′-GTTACTGCCCTGTGGGGCAA-3′; 5′-CAAAGGTGCCCTTGAGGTT-3′) was used to amplify the single-copy nuclear β-global gene. Relative mtDNA copy number was calculated as the ratio of mRNA level for the CYTB fragments relative to the β-global fragments.

Seahorse extracellular flux assay

Experimental design for seahorse assay was on the basis of Agilent Seahorse XF Cell Mito Stress Test Kit (Agilent Technologies). SMCs were plated and grown on Agilent Seahorse XF Cell Culture Microplate in F12K media containing 10% FBS and then were transfected with empty vectors (pcDNA-3.1), no-MTS DNMT1, or MTS-DNMT1. Cells were then rinsed and cultured in XF Base Medium in 37℃ incubator without CO_2 for 1 hour. Traces of mitochondrial oxygen consumption rates (OCR) of the cells were measured using a Seahorse XF24 flux analyzer, with sequential injections of mitochondrial effectors (Oligomycin, FCCP,

and Rotenone + antimycin A) at different time points. OCR was measured as the indicator of mitochondrial respiration.

Gel contraction assay

SMCs were pretreated at a density of 300,000 cells/ml and were suspended in the collagen solution, the collagen solution consist of Type I collagen (3.32 mg/mL), 0.1M NaOH, 2×F12K, and FBS mixed in the volume ratio of 33:5:50:12 (final concentration of type I collagen, 1.0 mg/ml). The cell suspension was seeded in 24-well plates (400μl per well), and placed in an incubator for at least 1 hour to polymerize, and then the gels were detached carefully from well surface using a syringe needle. F12K medium containing 2% FBS was added into the well to suspend and equilibrate the collagen-cell slice overnight. Areas of slices were monitored to indicate the extent of gel contraction.

Traction force microscopy (TFM)

To measure SMC contractility at single cell level, we implemented TFM. Briefly, polyacrylamide (PA) gel substrates were prepared by mixing acrylamide, bis-acrylamide, and fluorescence beads with a diameter of 0.2μm (Thermo Fisher), ammonium persulfate (APS) and tetramethylethylenediamine (TEMED) in ultrapure water. The mixture was added to the center of 20 mm diameter glass-bottomed dishes and then the gel surfaces were activated by incubation with sulfo-SANPAH (BioVision) and exposed to UV lights. The gels were then coated with fibronectin. The final gel stiffness was 9 kPa. SMCs were seeded on the gel substrates and then cultured for about 24h. A spatial map for each dish of fluorescent beads that were embedded within the gel substrate directly underneath the cells was taken by a fluorescence microscope (Leica DMI6000B). Following detachment of cells from the substrates using 0.5% trypsin, a second spatial map of the same beads was obtained. Monolayer displacement was calculated by comparing the two maps using a Fourier-based

difference-with-interpolation image analysis.[1] To characterize the contractile forces of each cell, the elastic strain energy stored in gels due to cell tractions was calculated as the product of local tractions and deformations, integrated over the spreading area of the cells.[2]

Mitochondria transplantation *in vitro* and *ex vivo*

Mitochondrial transplantation *in vitro* was performed as previously described.[3, 4] In brief, the donor cells were trypsinized and washed by buffer I (1 mmol/L Tris–HCl, 130 mmol/L NaCl, 5 mmol/L KCl, 7.5 mmol/L $MgCl_2$, pH 7.0), and then ruptured in 50% of the packed cell volume of buffer II (3.5 mmol/L Tris–HCl, 2.0 mmol/L NaCl, 0.5 mmol/L $MgCl_2$, pH 7.8) by using a glass homogenizer. The cell homogenate was quickly blended with 1:9 of the packed cell volume of buffer III (350 mmol/L Tris–HCl, 200 mmol/L NaCl, 50 mmol/L $MgCl_2$, pH 7.8) and centrifuged for 5 min at 1500g. The supernatant was then added into a new centrifuge tubes and centrifuged for 1 min at 15000 g. The mitochondria pellets were washed once by buffer IV (35 mmol/L Tris–HCl, 20 mmol/L NaCl, 5 mmol/L $MgCl_2$, pH 7.8), twice by buffer V (10 mmol/L Tris–HCl, 1 mmol/L EDTA, 320 mmol/L saccharose, pH 7.4), then resuspended in the appropriate incubation medium. The whole purification process was performed on ice or at 4°C. The recipient cells were grown to 70-80% confluence and were then cultured in F12 media supplemented with 110 μg/ml sodium pyruvate, 50 μg/ml uridine and 100 ng/ml ethidium bromide to remove endogenous mitochondria. Fourteen days later, mtDNA copy number and cell immunofluorescence were assessed in the recipient cells to verify the efficiency of mitochondria-removal. The recipient cells were then incubated with 2.35×10^8/ml of isolated mitochondria from control, PDGF-BB treated or MTS-DNMT1-transfected SMCs in an incubator at 37 °C for 24 hours. For mitochondria delivery *ex vivo*, mouse arterial rings (3mm) were incubated in mitochondrial suspensions (isolated mitochondria (2.35×10^8/ml) in DMEM medium) for 12 hours.

Isolation of mouse embryonic fibroblasts (MEFs)

The MEFs were isolated from the embryo of Dnmt1$^{flox/flox}$ mice. In brief, mice were sacrificed when the embryos are 13.5-14.5 days old. The uterine horns containing embryos were removed from mouse and were placed in dish of sterile PBS on ice. Then the embryos were moved from uterine by using sharp forceps and minced by scissor. The mince was transfered to 15 mL conical flask containing 5 mL 0.1% trypsin and was pipetted up and down several times to break up tissue chunks and get cells into suspension. We repeated digestion process up to 4 times to resuspend all cells and then the cells were centrifuged at 1000 rpm. The MEF sediments were cultured in DMEM medium with L-glutamine.

Terminal Deoxynucleotidyltransferase-mediated dUTP Nick End Labeling (TUNEL) apoptosis analysis

TUNEL apoptosis analysis was performed using the Meilum One Step TUNEL assay kit (TRITC) according to the manufacturer's instructions. In each biological repeat, at least 10 random microscopy fields were counted. The results are expressed as the percentage of TUNEL-positive stained cells (Apoptosis index = (TUNEL positive cells/total number of cells) X 100).

Lactate Dehydrogenase (LDH) Measurement

Conditioned media were collected from cells with the indicated treatments. The LDH concentration in the media was spectrophotometrically assayed using a Lactate Dehydrogenase Assay kit (Shanghai Gensource) according to the manufacturer's instructions.

Experimental animals

All animal studies were performed in accordance with the approved protocol (LA2015017) of the Animal Care and Use Committee of Peking University and were performed in accordance with the "Guide for the care

and use of laboratory animals" published by the US National Institutes of Health (publication No. 85–23, revised 1996). 8-week-old C57/BL6 wildtype mice were obtained from the Experimental Animal Center at Peking University Health Science Center (Beijing, China). Dnmt1$^{flox/flox}$ (B6.129S4-Dnmt1^{tm2Jae}/Mmucd, stock number 014114-UCD) mice were obtained from the Mutant Mouse Resource and Research Center (MMRRC) at the Jackson Laboratory. Animals were kept in specific pathogen-free cages, 12-hour light-dark cycle, controlled temperature and humidity, and had water and food ad libitum. Anesthetization and euthanasia were performed by intraperitoneal injection of sodium pentobarbital (50mg/kg and 150mg/kg, respectively). Anaesthetic depth was tested with multiple toe pinches and response by the animals indicated the necessity for supplementary anaesthesia administration, resulting in an average dose of 75 mg/kg sodium pentobarbital.

Animal models

For the complete ligation model, the left carotid bifurcation of mice was exposed following a neck incision, and the proximal part of the common left carotid artery was ligated with a 6-0 silk suture. The right carotid arteries were served as control. Mice were sacrificed and both sides of carotid arteries were isolated 1 and 4 weeks after ligation to be subjected to MSP assay or morphological observation, respectively. Vessels were dissected out for DNA/RNA extraction or embedding in Tissue-Tek OCT compound after perfused with a fixative (4% paraformaldehyde in PBS) or PBS. For the mouse model of carotid artery injury by a guide wire, the left carotid bifurcation of mice was exposed following a neck incision, and the left common carotid artery and internal carotid artery were clamped with forceps, the external carotid artery was ligated with a 6-0 silk suture and was incised at the proximal part. The guide wire was inserted into the internal carotid artery and rubbed the intima 5 times repeatedly. The right carotid arteries were served as control. At 4 weeks after operation, the mice were sacrificed and the vessels were dissected out for embedding in Tissue-Tek OCT compound or DNA extraction after perfused with a fixative (4% paraformaldehyde in PBS) or PBS. Vessels

for MSP assay were subjected to mechanical rubbing to achieve de-endothelialization.

Tissue preparation, hematoxylin-eosin (H&E) staining, and immunofluorescence

Mouse carotid arteries were prepared in frozen sections. Human internal mammary arteries and endarterectomy specimens were prepared in paraffin sections. Sections from the frozen blocks were stained with hematoxylin and eosin in turn, and the sections were washed by running water and immersed into ethanol at a concentration gradient from 95%-100%, and then the sections were dehydrated by xylene. The sections were visualized by a light microscopy.

For immunofluorescence assay, frozen sections were re-fixed by acetone and permeabilized by Triton X-100. Paraffin sections were dewaxed and then antigen repairment was performed. Nonspecific binding was blocked by 3-5% BSA in PBS for 1 hour and then immunofluorescence was performed on frozen or paraffin sections using the following antibodies at a 1:100 dilution: DNMT1 (Santa Cruz Biotech), SMα-actin (Santa Cruz Biotech) and DsRED2 (Origene) overnight at 4°C. The sections were incubated with secondary antibodies including Alexa Fluor 488-conjugated donkey/goat anti-rabbit/-mouse IgG (1:500, ThermoFisher) or Alexa Fluor 555-conjugated donkey anti-rabbit/-goat IgG (1:500, ThermoFisher). Nuclei were counterstained with DAPI. The sections were then visualized by fluorescence confocal microscopy (Leica TCS SP8).

Human specimens

Endarterectomy specimens and internal mammary arteries were obtained from patients undergoing coronary artery bypass grafting or with carotid occlusive diseases. All samples were obtained with the agreement of the patients and approved by the Peking University People's Hospital Medical Ethics Committee (2015PHB024). The experiments using human specimens were carried out in accordance with the approved guidelines. All human studies described in this work conform to the principles outlined in the Declaration of Helsinki.

Arterial respiration

Oxygraph-2k (O2k; OROBOROS Instruments) was used for measuring mitochondria respiration in mouse arteries. Mouse common carotid arteries were dissected and maintained in BIOPS (10 mmol/L Ca-EGTA buffer, 0.1 μmol/L free calcium, 20 mmol/L imidazole, 20 mmol/L taurine, 50 mmol/L MES, 0.5 mmol/L dithiothreitol, 6.56 mmol/L $MgCl_2$, 5.77 mmol/L ATP and 15 mmol/L phosphocreatine, pH7.1) at 4°C. Samples were permeabilized in 50 μg/ml saponin solution at 4°C for 1 hour and were then washed with MiR05 buffer (0.5 mmol/L EGTA, 3 mmol/L $MgCl_2 \cdot 6H_2O$, 60 mmol/L K-lactobionate, 20 mmol/L taurine, 10 mmol/L KH_2PO_4, 20 mmol/L HEPES, 110 mmol/L sucrose, 1g/L fatty acid free BSA, pH 7.1) before being put into the electrode chambers.

Substrates and inhibitors were added sequentially to determine complex I, II, and IV respiration as indicated in a previous study.[5] Complex I-supported respiration rates were measured by using 10 mmol/L glutamate + 5 mmol/L malate. 5 mmol/L ADP was then added to stimulate State 3 respiration. After the addition of 1 μmol/L rotenone for the inhibition of complex I, complex II-supported respiration was assessed with 10 mmol/L succinate. Next, Complex III-supported respiration was inhibited by 5 μmol/L antimycin, and then 0.5 mmol/L N,N,N′,N′-Tetramethyl-p-phenylenediamine (TMPD) + 2 mmol/L ascorbate were used to induce complex IV-supported respiration. The intactness of the outer mitochondrial membrane was assessed by adding 10 μmol/L cytochrome C finally. The platform oxygen flux (pmol/s) difference was measured by Oxygraph-2k after every reagent was added into chambers. Vessels were removed from the electrode chambers and dried, with oxygen flux expressed as picomoles O_2 per second per dry weight (pmol/s/mg).

Wire myograph

Wire myograph experiment was performed as previously described.[6] In brief, Mice were euthanized and

common carotid arteries were harvested. The arteries with or without mitochondrial transplantation were placed in ice-cold Krebs solution (119 mmol/L NaCl, 4.7 mmol/L KCl, 2.5 mmol/L $CaCl_2$, 1 mmol/L $MgCl_2$, 25 mmol/L $NaHCO_3$, 1.2 mmol/L KH_2PO_4, 11 mmol/L glucose) with oxygen. Common carotid arteries were cleaned of adhering tissue and cut into ring segments of 3mm in length, and then the ring segments were suspended in the myograph (Danish Myo Technology, Aarhus, Denmark). To monitor the changes in isometric tension, KCl-simulated and phenylephrine-simulated vascular contraction were expressed as active tension. We set the contraction baseline at about 1.5mN, the active tension was the difference value between simulated contraction and basal contraction per unit ring length (mN/mm).

Cell proliferation assay

Standard fluorescent immunocytochemistry was carried out to exam cell proliferation, as reflected by positive staining of proliferative marker, Ki67. The numbers of Ki67-positive-stained cells and total cells (DAPI-positive cells) were scored in 10 randomly selected microscopic fields and then the Ki67-positive cell ratios were calculated.

Intracellular real-time Ca^{2+} imaging

Real-time quantification of intracellular cytosolic Ca^{2+} ([Ca^{2+}]i) was detected using fura-4 AM (Invitrogen), a Ca^{2+}-sensitive radiometric fluorescent dye. SMCs were grown on dishes with glass bottom (NEST), and incubated with 5 μmol/L fura-4 AM in F12K medium for 30 minutes in an incubator. Real-time concentration of [Ca^{2+}]i was visualized by confocal microscopy (Leica TCS SP8). The cells were stimulated by 60 mmol/L potassium at 1 minute time point and were monitored for 7 minutes. Maximum [Ca^{2+}]i was measured at a period of 90 seconds. Mean Ca^{2+} flux response was calculated from all the cells present in microscopic fields from at least 3 separated experiments.

Statistical analysis

Data are expressed as mean±SEM from at least 3 independent experiments. Results were analyzed by SPSS and GraphPad Prism 7.0 software for statistical significance between treatment groups. Normality and equal variance tests were confirmed before further analysis. Parametric data with equal variance were analyzed by paired or unpaired 2-tailed Student's t test. Nonparametric data were analyzed using Mann-Whitney U test. Multiple comparisons of means were analyzed using 1-way ANOVA followed by Tukey's post hoc test. Values of $P < 0.05$ were considered statistically significant.

REFERENCES

1. Metzner, C., Raupach, C., Zitterbart, D.P. & Fabry, B. Simple model of cytoskeletal fluctuations. *Phys Rev E Stat Nonlin Soft Matter Phys* 76, 021925 (2007).
2. Butler, J.P., Tolic-Norrelykke, I.M., Fabry, B. & Fredberg, J.J. Traction fields, moments, and strain energy that cells exert on their surroundings. *Am J Physiol Cell Physiol* 282, C595-605 (2002).
3. Zhu, L. et al. Mitochondrial transplantation attenuates hypoxic pulmonary hypertension. *Oncotarget* 7, 48925-48940 (2016).
4. Zhou, J. et al. Mitochondrial transplantation attenuates hypoxic pulmonary vasoconstriction. *Oncotarget* 7, 31284-31298 (2016).
5. Yu, E.P.K. et al. Mitochondrial Respiration Is Reduced in Atherosclerosis, Promoting Necrotic Core Formation and Reducing Relative Fibrous Cap Thickness. *Arterioscler Thromb Vasc Biol* 37, 2322-2332 (2017).
6. Liu, L. et al. Protein kinase Cdelta contributes to phenylephrine-mediated contraction in the aortae of high fat diet-induced obese mice. *Biochem Biophys Res Commun* 446, 1179-1183 (2014).
