# Supplementary material for: Hypermethylation of mitochondrial DNA in vascular smooth muscle cells impairs cell contractility
Source: Cell Death Dis. 2020 Jan 20;11(1):35. doi: 10.1038/s41419-020-2240-7 (PMC6971246; doi:10.1038/s41419-020-2240-7)
Supplement: Supplementary file 17 — DECLARATION OF CONTRIBUTIONS TO ARTICLE [file 41419_2020_2240_MOESM17_ESM.pdf]

DECLARATION OF CONTRIBUTIONS TO ARTICLE

ADMC

Manuscript Number: CDDIS-19-3345RRR

Journal Name: *Cell Death & Disease* (the 'Journal')

Proposed Title of the Contribution:

Contribution to the manuscript entitled Hypermethylation of Mitochondrial DNA in Vascular Smooth Muscle Cells Impairs Cell Contractility (the 'Contribution)

Author(s):

Yue-Feng Liu, Juan-Juan Zhu, Xiao Yu Tian, Han Liu, Tao Zhang, Yun-Peng Zhang, Si-An Xie, Ming Zheng, Wei Kong, Wei-Juan Yao, Wei Pang, Chuan-Rong Zhao, Yuan-Jun Tang, Jing Zhou (the 'Authors')

For all *CDDis* articles, each person named as an author in the published version must be able to show he or she has contributed substantially to the article.

Authorship credit should be based on 1) substantial contributions to conception and design, acquisition of data, or analysis and interpretation of data; 2) drafting the article or revising it critically for important intellectual content; and 3) final approval of the version to be published. Authors should meet conditions 1, 2 and 3.

Any person who cannot be shown to have made a substantial contribution to the article cannot be listed as an author in the final version. The name of any person who is deemed to have made a minor contribution can, however, appear in the Acknowledgments section of the article.

Please complete the table below to indicate the contributions of all named authors to the manuscript.

Author Full Name:	Specification of Contribution to the Manuscript:
Jing Zhou	designed the study and drafted/revised the manuscript
Yue-Feng Liu	designed the study, conducted most of the experiments, collected and analysed the data, and help to write the manuscript
Juan-Juan Zhu	performed many of the experiments, conducted data analysis, revised the manuscript
Xiao-Yu Tian	provided scientific input and strategically planned the experiments, made a substantial contribution to the part of smooth muscle cell contration
Han-Liu	performed many of the experiments and conducted data analysis
Tao Zhang	contributed to experiments by providing clinical and human specimen
Yun-Peng Zhang	performed experiments and conducted data analysis, made a substantial contribution to the part of ChIP and MSP experiments
Si-An Xie	performed experiments and conducted data analysis, made a substantial contribution to the part of gel contraction assay and traction force microscopy experiments
Ming Zheng	provided scientific input and strategically planned some of the experiments, made a substantial contribution to the part of mitochondrial experiments
Wei Kong	provided scientific input and strategically planned the experiments, made a substantial contribution to the part of mitochondrial transplantation experiments
Wei-Juan Yao	provided scientific input and strategically planned the experiments, provided a lot of improvement measure to the experiments
Wei Pang	provided scientific input and strategically planned the experiments
Chuan-rong Zhao and Yuan-jun Tang	performed some of the experiments and conducted data analysis

Please complete the table below to indicate the contributions of all named authors to the figures.

Figure 1:

YFL generated the data and images and prepared panels A, B, C, D, H, I, J, and K. JJZ and HL analyzed the data and prepared panels E, F, and G. YPZ designed the MSP primers and direct the MSP and ChIP experimental technique (panels I,J,K).

Figure 2:

YFL generated the data and images and prepared panels A, B, C, D, E, F, G, and H. MZ advised on the mitochondrial respiration experiment and analyzed the data. YFL, HL and YJT performed the mitochondrial respiration experiment and prepared panels I and J.

Figure 3:

YFL generated the data and images and prepared panels A, B, C, D, E, F, G, H, I, and J. SAX direct the experimental technique and help to prepare panels B, C, D, E, F, G and H. WJY and WP advised on the all contraction experiment.

Figure 4:

YFL generated the data and images and prepared panels A, B, C, D, E, F, and G. WK and YJT advised on the mitochondrial transplantation experiment analyzed the data. SAX direct the experimental technique and help to prepare panels D, E, F, and G.

Figure 5:

YFL generated the data and images and prepared panels A, B, C, D, E, F, and G. JJZ and CRZ help to carry out the animal experiments of panels B and D. TZ provided the valuable clinical patients samples of the panels F and G, and perform the immunofluorescence experiment (supplementary data).YPZ designed the MSP primers and direct the MSP experimental technique (panels C,E,F and G)

Figure 6:

In Fig 6, YFL generated the data and images and prepared panels B, C, D, E, F, G and H. HL prepared panel A. JJZ, HL and CRZ helped with the animal experiment and prepared the sections. MZ advised on the arterial complex respiration experiment. XYT advised on the myograph experiment and analyzed the data. SAX and YJT helped with the myograph experiment.
In Fig 7, YFL generated the data and images and prepared panels A, B, C, D, and E. XYT advised on the myograph experiment and analyzed the data. JJZ help to prepared panel A.

Signed for and on behalf of the Author(s):	Print Name:	Date:
	Jing Zhou	2019-12-9
