# Supplementary material for: Hypermethylation of mitochondrial DNA in vascular smooth muscle cells impairs cell contractility
Source: Cell Death Dis. 2020 Jan 20;11(1):35. doi: 10.1038/s41419-020-2240-7 (PMC6971246; doi:10.1038/s41419-020-2240-7)

Entire Unedited Gel

Full unedited gel for Figure 1A

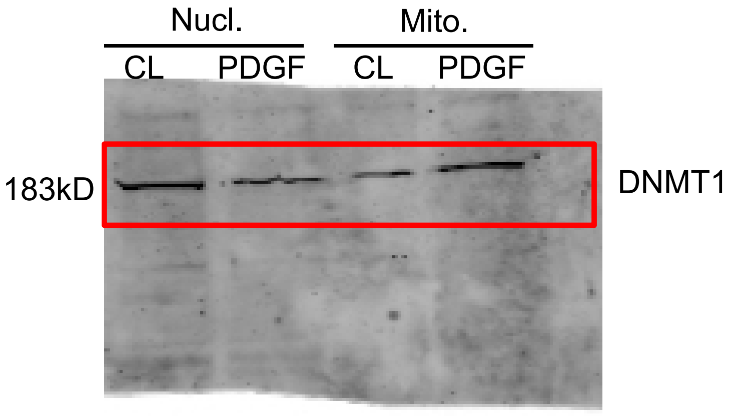

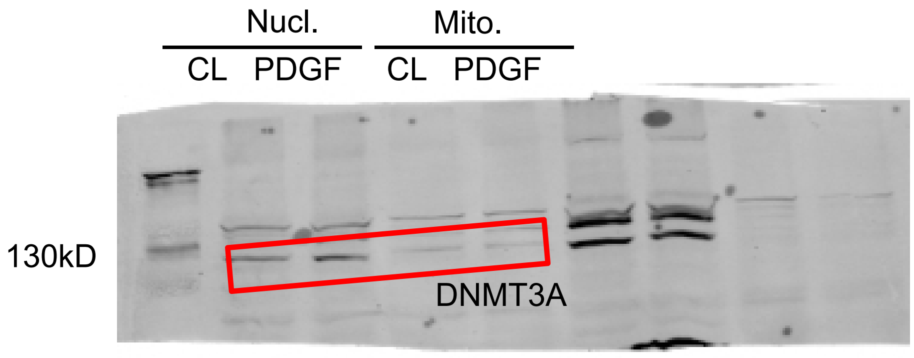

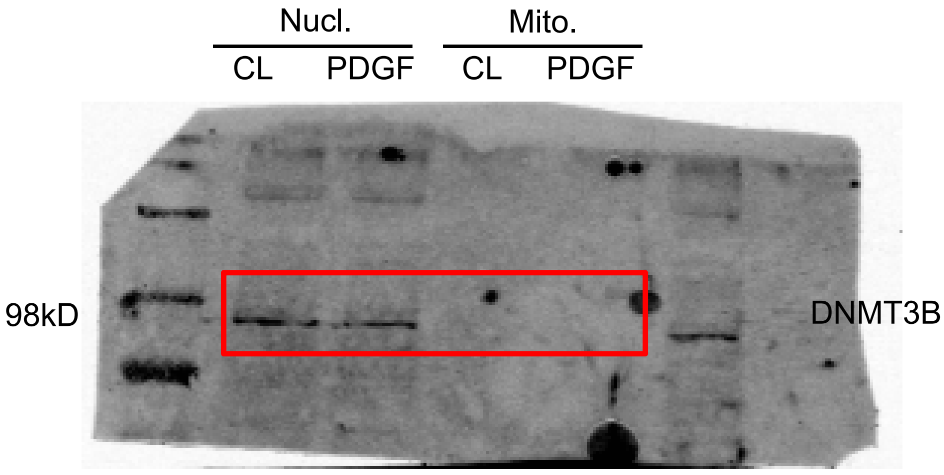

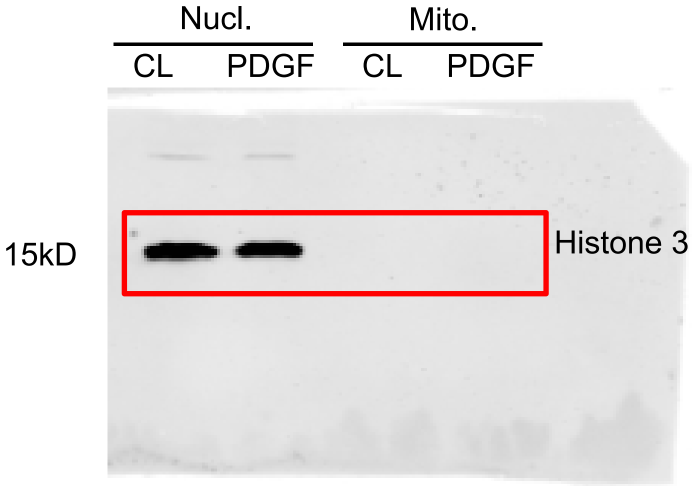

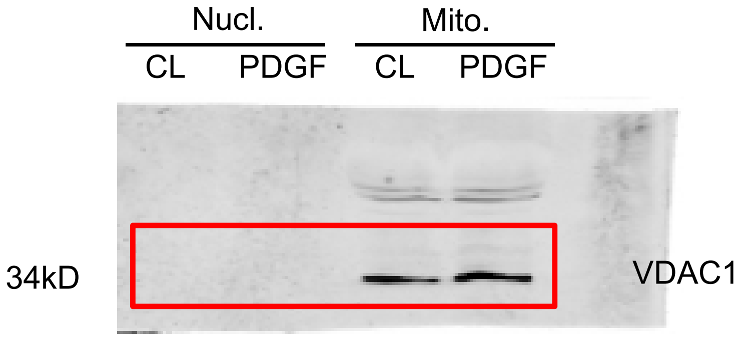

Full unedited gel for Figure 1K

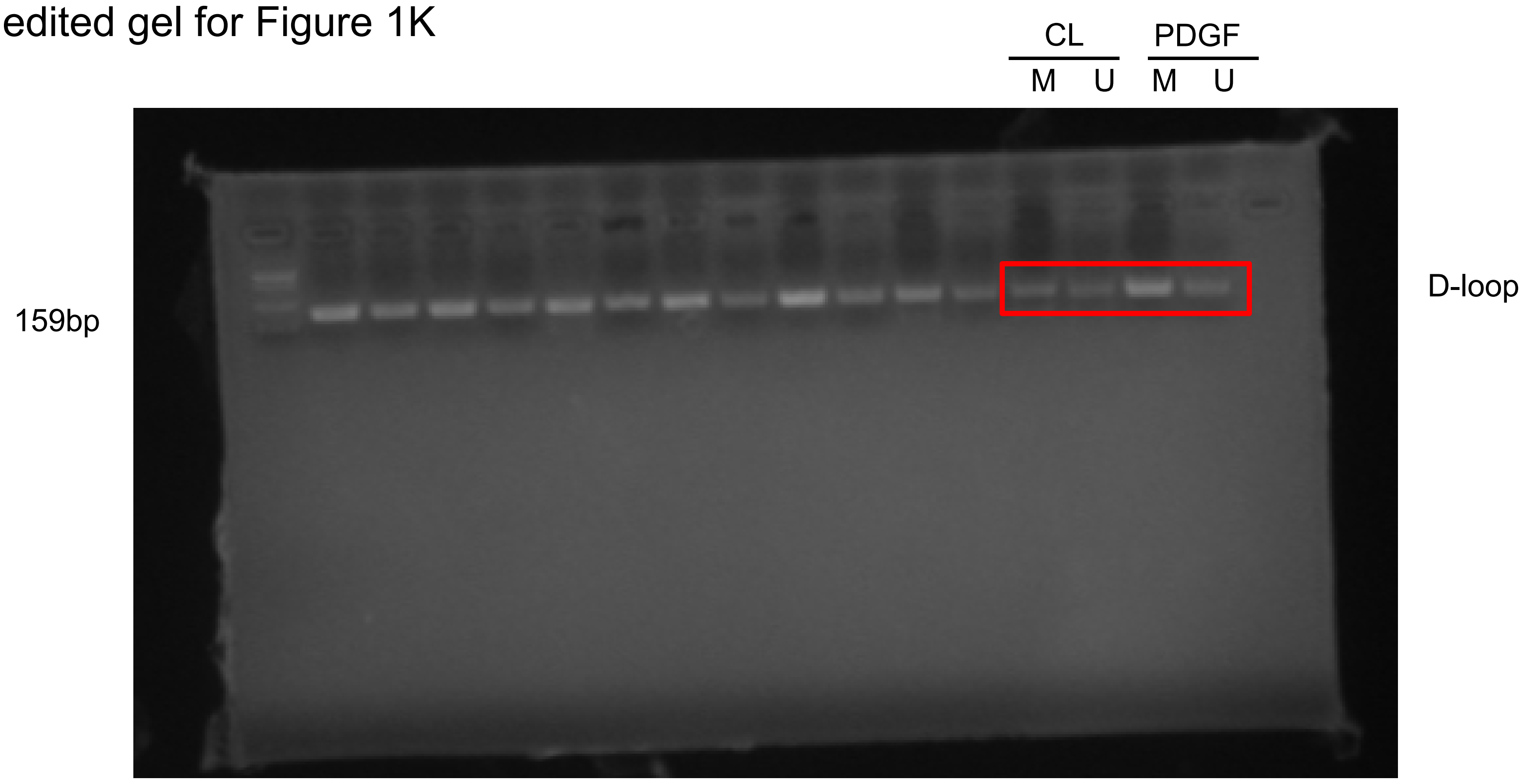

Full unedited gel for Figure 2C

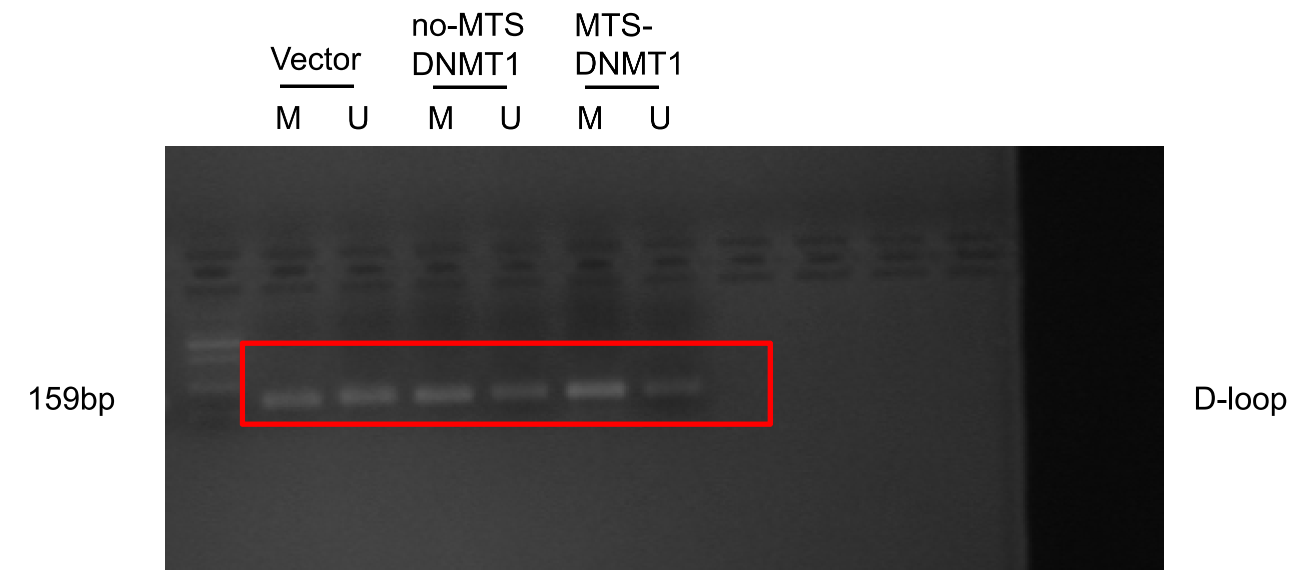

Full unedited gel for Figure 3I

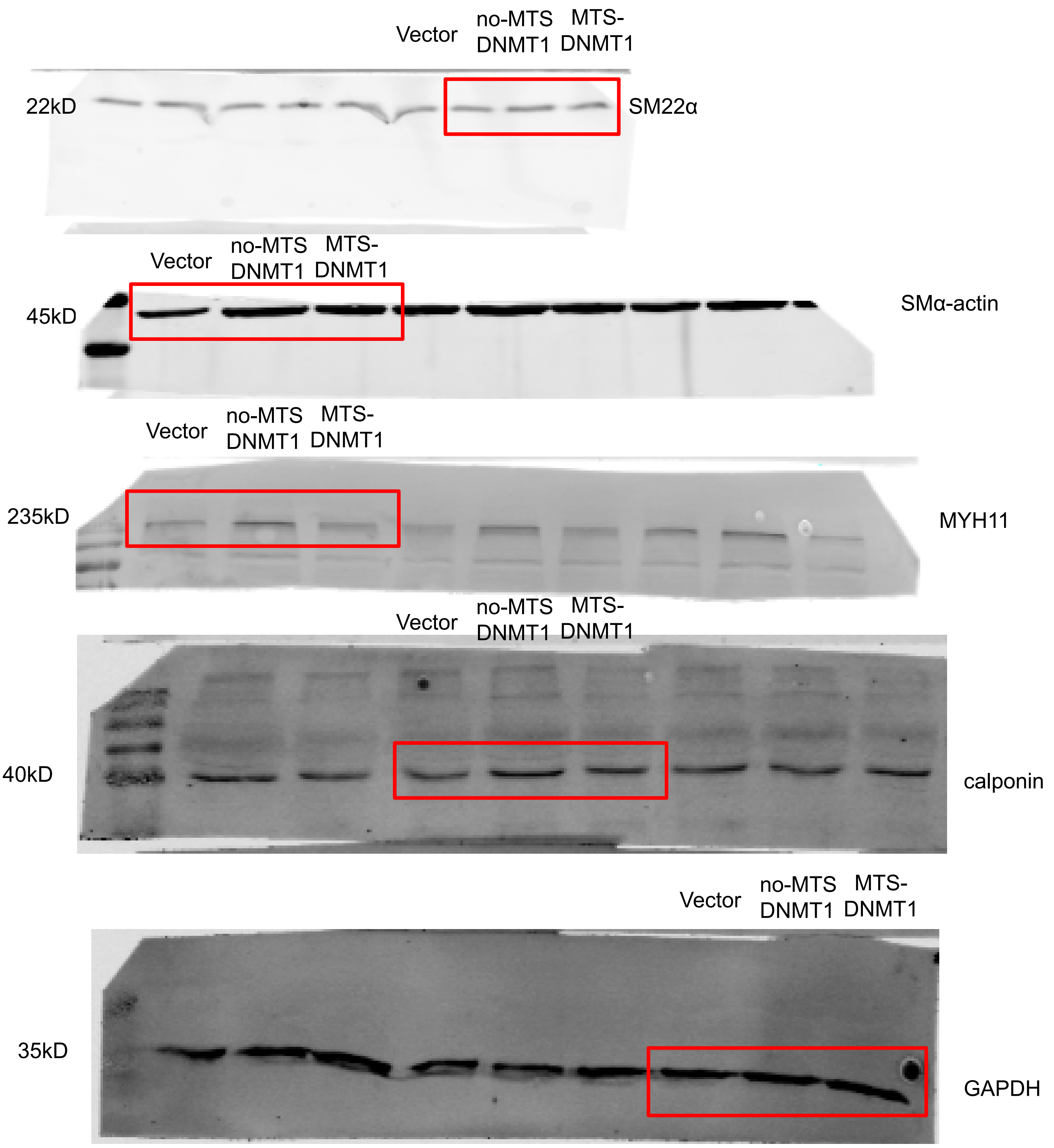

Full unedited gel for Figure 5C

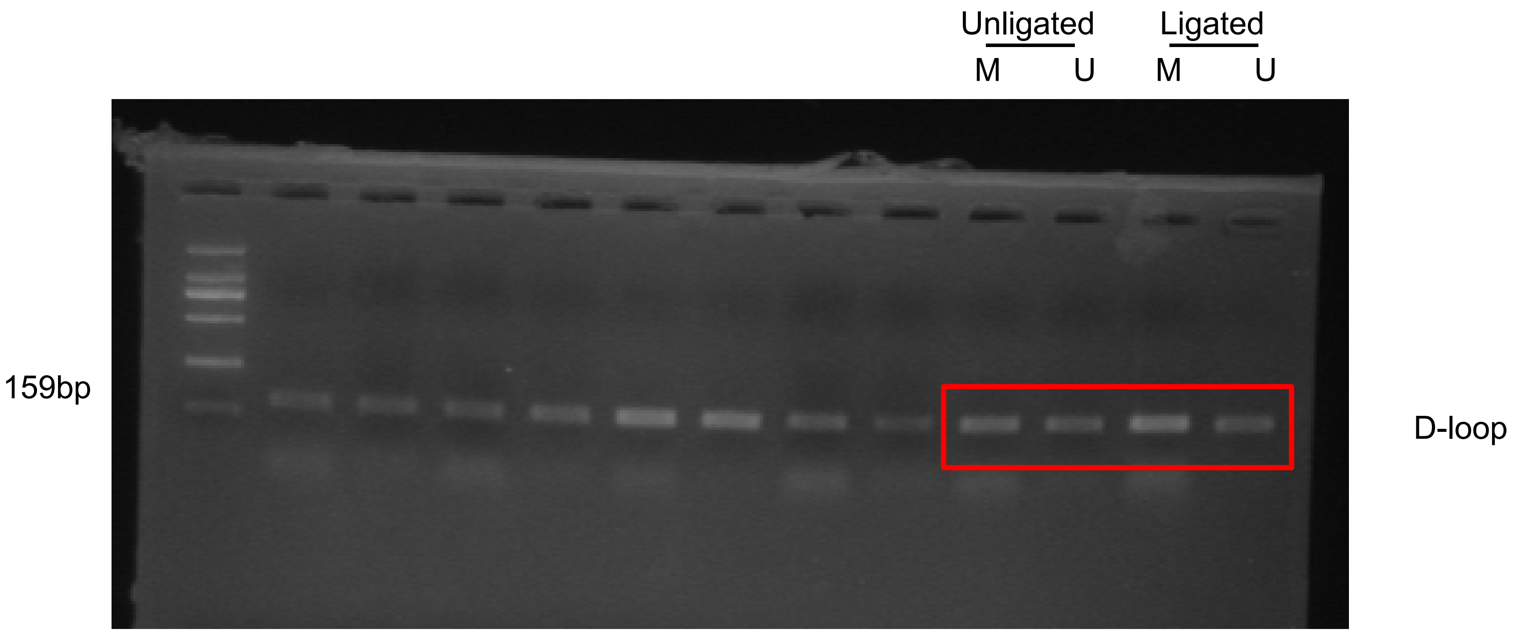

Full unedited gel for Figure 5E

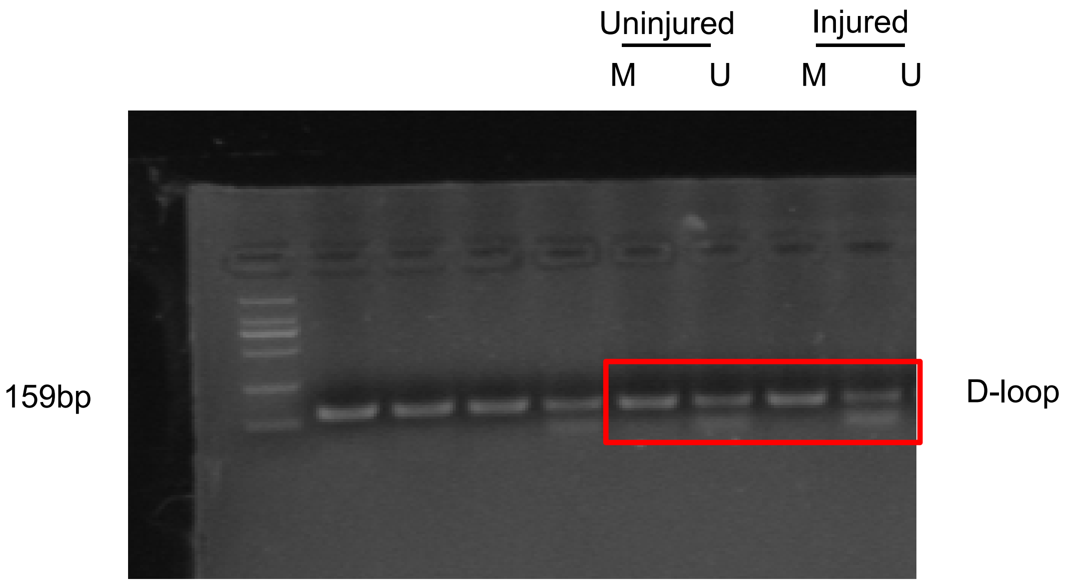

Full unedited gel for Figure 5F

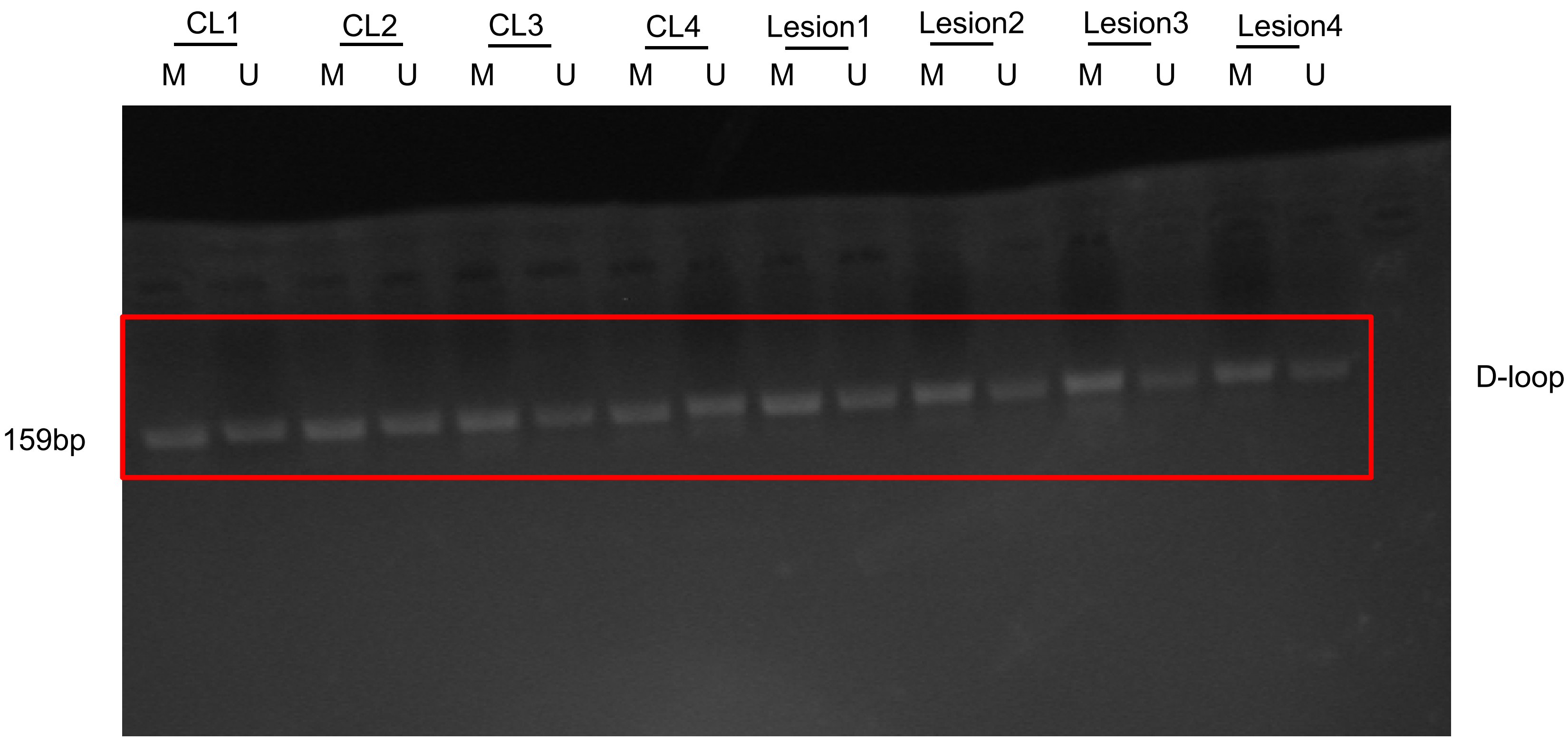

Full unedited gel for Figure 5G

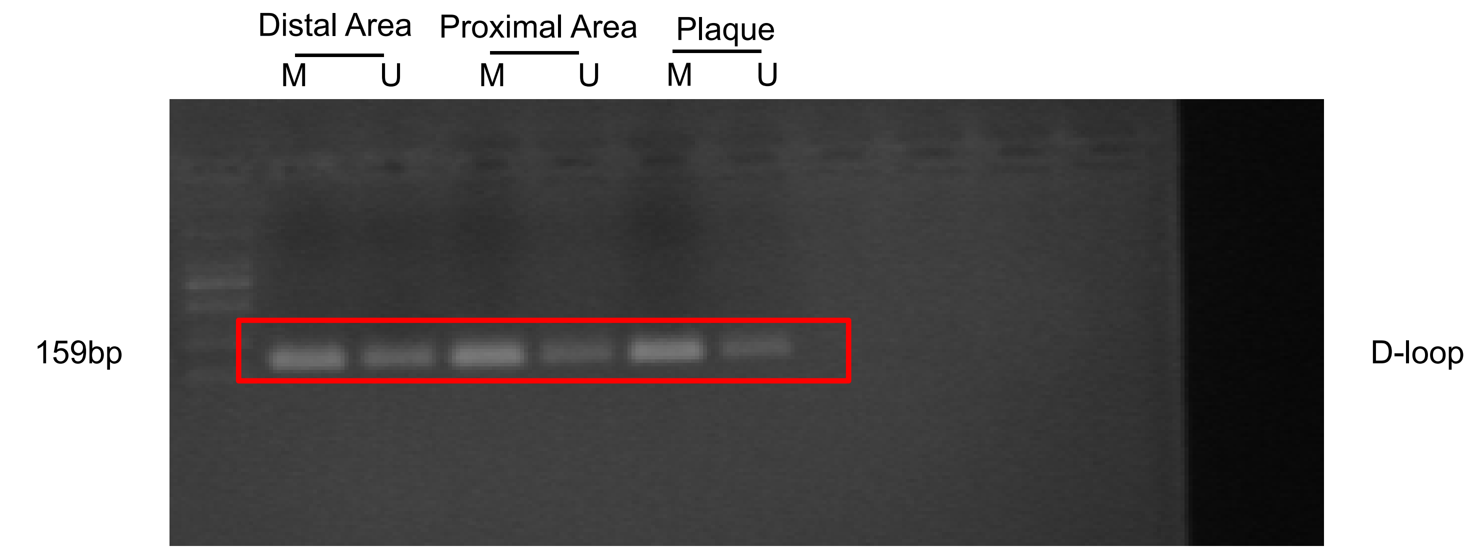

Full unedited gel for Figure S1A

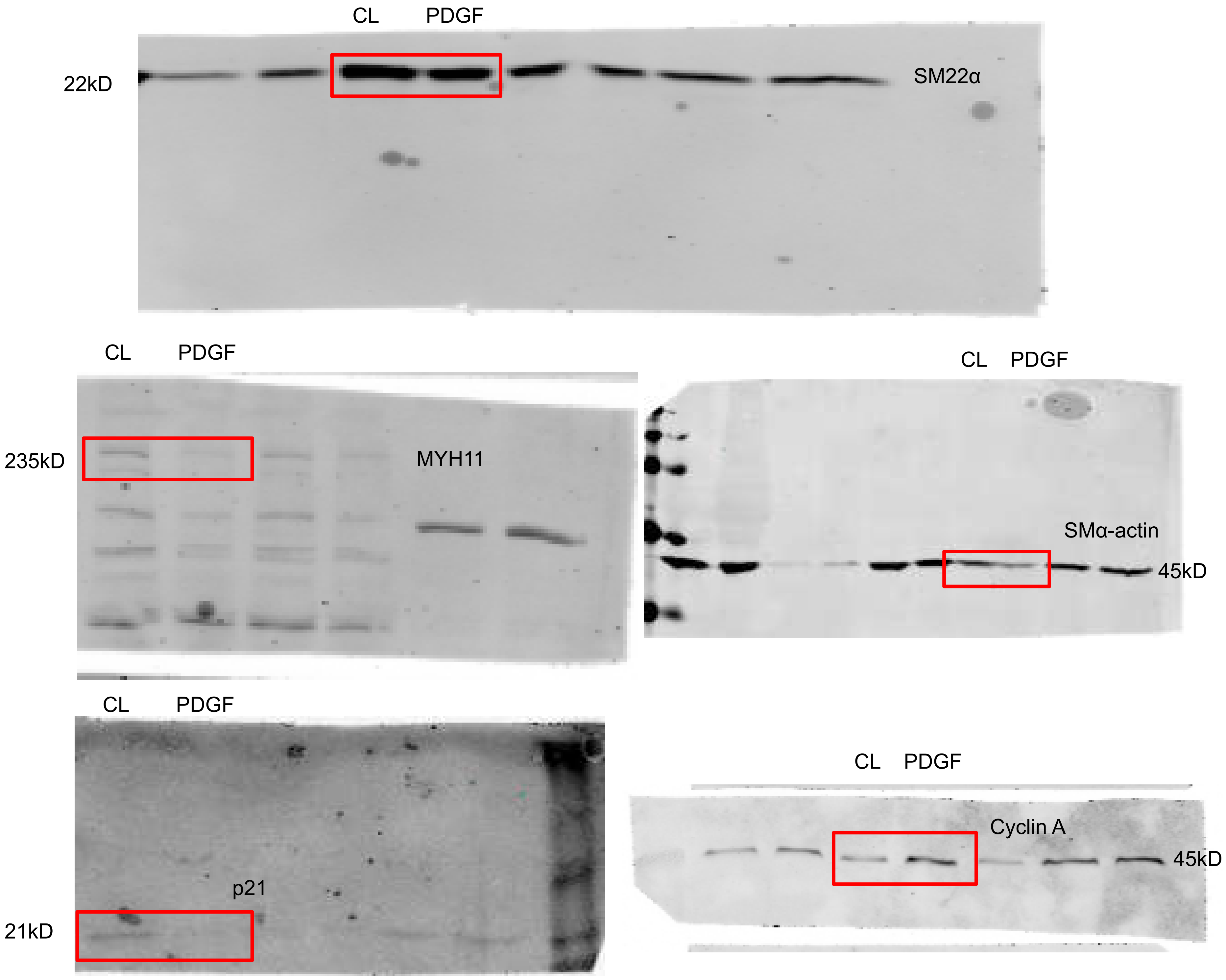

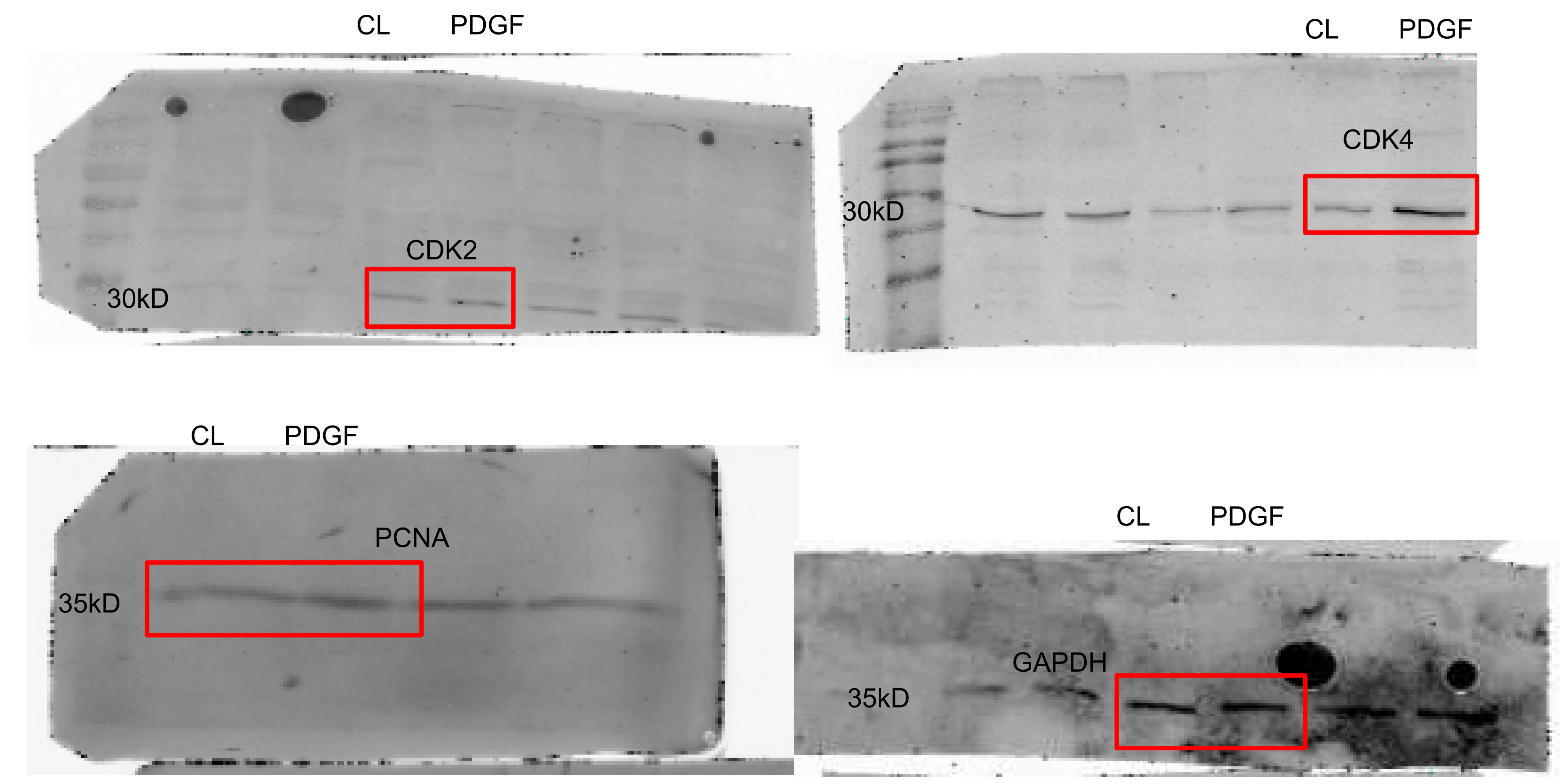

Full unedited gel for Figure S2A

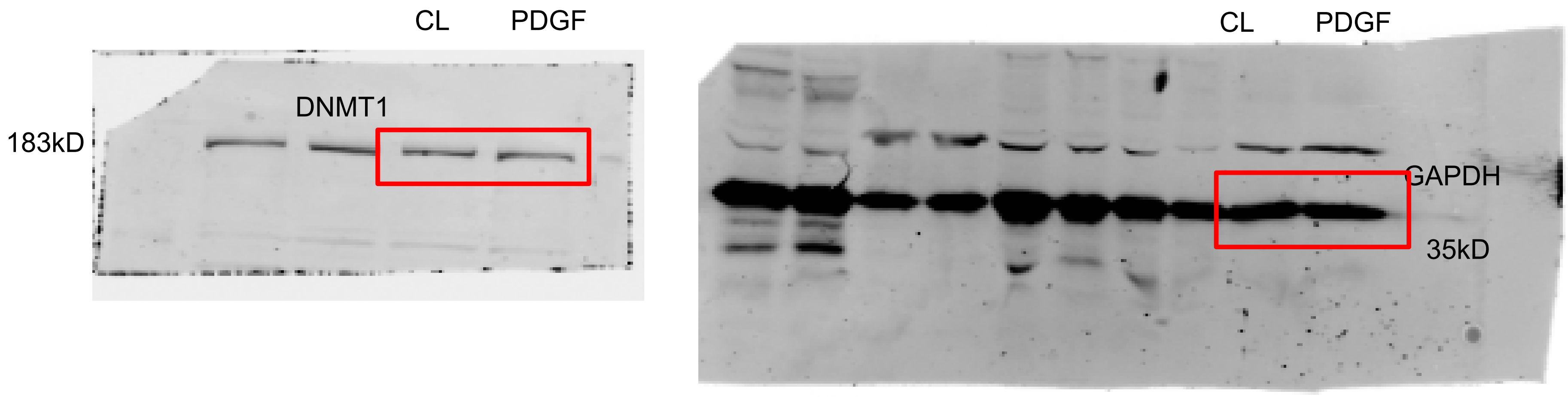

Full unedited gel for Figure S3

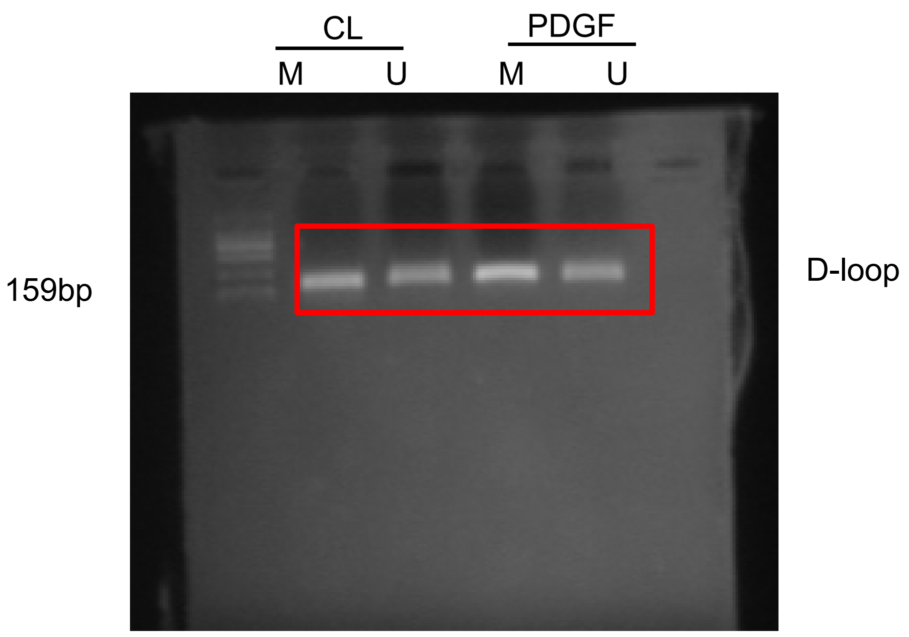

Full unedited gel for Figure S4

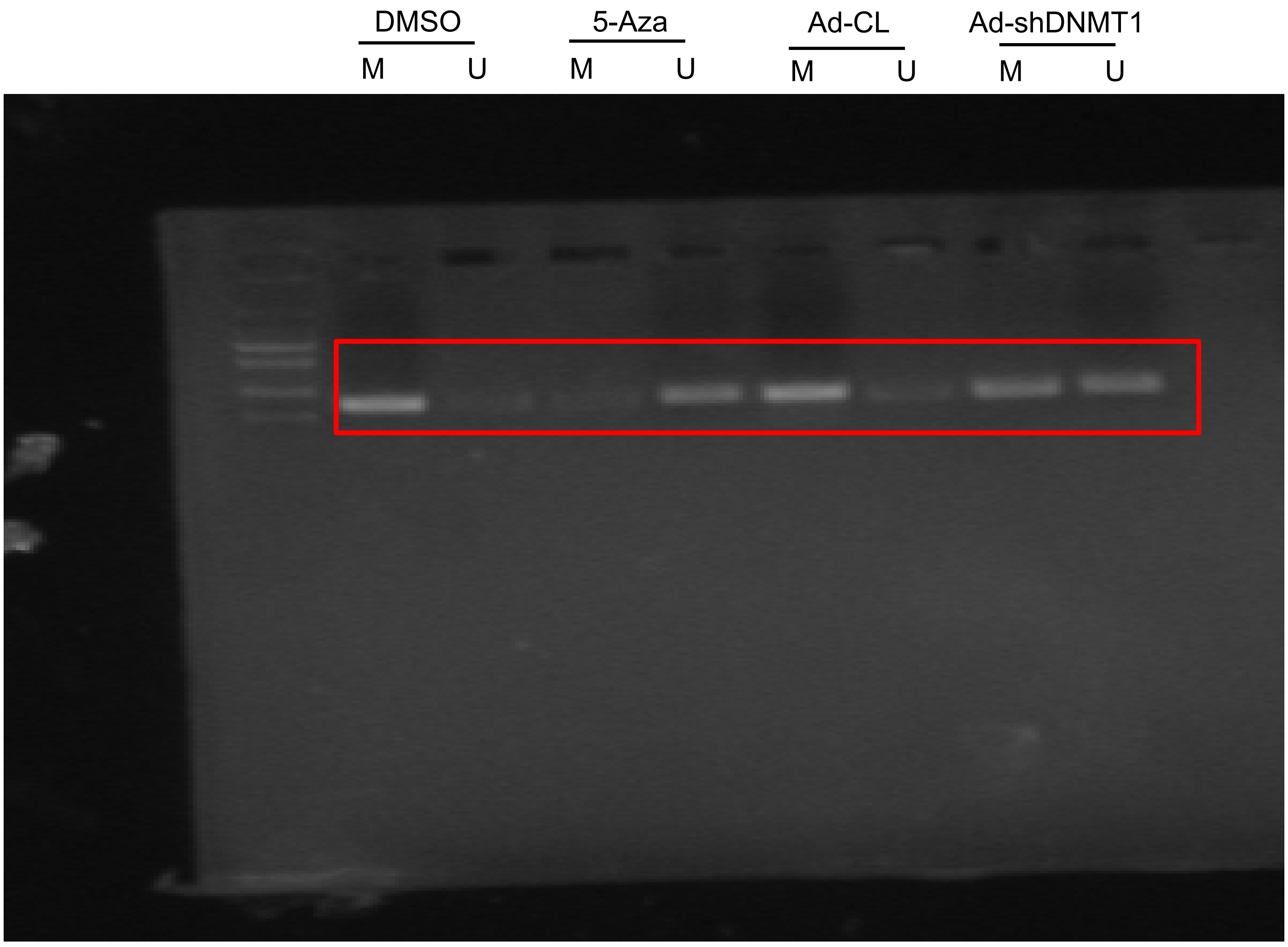

Full unedited gel for Figure S12A

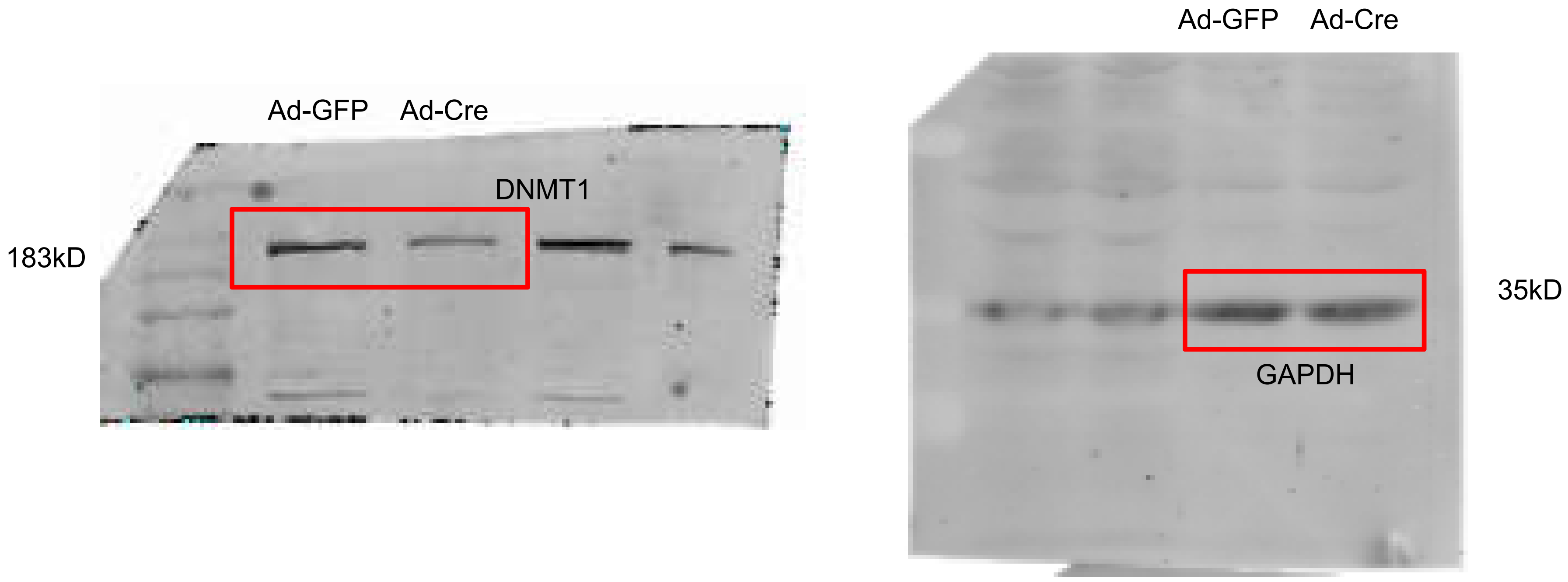

Supplement: Supplementary file 18 — Entire Unedited Gel [file 41419_2020_2240_MOESM18_ESM.pdf]
